# A Randomized Controlled Trial to Evaluate the Effects of Full-Fat and Low-Fat Fermented Dairy Foods on Markers of Cardiometabolic Health in Adults with Overweight or Obesity and Elevated Fasting Triglycerides

**DOI:** 10.1016/j.cdnut.2026.109516

**Published:** 2026-08-14

**Authors:** Kevin C Maki, Nana Gletsu-Miller, Indika Edirisinghe, Meredith L Wilcox, Caryn G Adams, Orsolya M Palacios, Lisa A Spence, Karen L Sprague, Emma James, Allon N Friedman, Elizabeth Guzman, Britt Burton-Freeman

**Affiliations:** 1 Midwest Biomedical Research, Addison, IL, United States; 2 Department of Applied Health Science, Indiana University School of Public Health-Bloomington, Bloomington, IN, United States; 3 Nutrition and Exercise Research Center, Indiana University School of Public Health-Bloomington, Bloomington, IN, United States; 4 Center for Nutrition Research, Department of Food Science and Nutrition, Illinois Institute of Technology, IL, United States; 5 Department of Medicine, Indiana University School of Medicine, Indianapolis, IN, United States

**Keywords:** carbohydrate metabolism, cardiometabolic health, dairy, inflammation, lipid metabolism

## Abstract

**Background:**

Consumption of fermented dairy has been associated with favorable cardiometabolic outcomes, but limited data from clinical trials have been available regarding the effects of fermented dairy products at differing levels of dairy fat on biomarkers of cardiometabolic health.

**Objectives:**

This randomized, controlled, parallel-arm, 12-wk trial evaluated the effect of replacing nondairy foods with 2 servings/d of either full-fat or low-fat fermented dairy foods on cardiometabolic health biomarkers in adults at risk of adverse cardiometabolic outcomes.

**Methods:**

Adults with a body mass index (BMI) of 25.0 to 39.9 kg/m^2^ and elevated fasting triglycerides (≥120 mg/dL) were randomly assigned to consume study foods provided as either 2 servings/d of full-fat fermented dairy foods, 2 servings/d of low-fat fermented dairy foods, or 2 servings/d of nondairy control foods, for 12 wk. Biomarkers of glucose homeostasis, lipid metabolism, inflammation, as well as anthropometrics and vital signs were assessed.

**Results:**

Mean (standard deviation) age and BMI of the intention-to-treat population (*N* = 110) were 45.7 (13.5) y and 31.3 (4.28) kg/m^2^, respectively. Full-fat fermented dairy showed a difference (*P* = 0.047) for change in least squares geometric mean waist circumference compared with control (−1.62% compared with 2.21%, respectively) with an effect estimate of −4.13% (−7.33, −0.81). No other statistically significant differences were observed, including the primary outcome of Matsuda index of insulin sensitivity [effect estimates of 5.24% (−20.0, 38.5) for low-fat fermented dairy and −3.11% (−26.3, 27.3) for full-fat fermented dairy, respectively, compared with control for difference in percent change in least squares geometric mean].

**Conclusions:**

Consumption of full-fat fermented dairy foods for 12 wk was associated with a small reduction in waist circumference compared with nondairy foods. No other statistically significant effects of 2 servings daily of low-fat or full-fat dairy were observed for other biomarkers of cardiometabolic health.

This trial was registered at clinicaltrials.gov as NCT04999462.

## Introduction

Cardiometabolic diseases, including atherosclerotic cardiovascular disease and type 2 diabetes (T2D), remain a major public health concern in the United States and throughout the world [1]. It is well accepted that dietary patterns play a critical role in cardiometabolic health, and intake of certain dairy foods has been associated with reduced risks for some cardiometabolic diseases [[2], [3], [4], [5], [6]]. The Dietary Guidelines for Americans (DGA) recommends intake of 3 daily servings of dairy foods without added sugars [7], and mean United States adult intake (1.47 servings/d) is well below the recommendation [8]. Although prior DGA have recommended low-fat or fat-free dairy foods out of concern for saturated fat intake [9], the most recent DGA directly challenges this decades-held view and advises the intake of whole milk-based, full-fat dairy foods with no or minimal processing [7]. This advice is based on the Scientific Foundation for the Dietary Guidelines for Americans report, which concludes that there is “remarkable lack of evidence” for recommending the replacement of whole-fat dairy with low-fat or fat-free versions [10]. Furthermore, the report states that dairy fat removal in products requires manufactured chemicals and other additives as a compensatory replacement [10], and the new DGA highlight dairy as an “excellent source of healthy fats, among other nutrients” [7].

Results from observational studies support the notion that the type of dairy food, along with the fat content and fatty acid composition, may influence the relationships between intakes of dairy foods and cardiometabolic outcomes [3,5,[11], [12], [13], [14], [15]]. Additionally, higher circulating levels of certain fatty acids found in dairy, including pentadecanoic acid (15:0), margaric acid (17:0), and *trans-*palmitoleic acid (*trans*-16:1n*–*7), have been associated with lower risks for metabolic syndrome, T2D, and atherosclerotic cardiovascular disease outcomes [[15], [16], [17]]. Meta-analyses of cohort study results suggest that consumption of fermented dairy foods, such as yogurts and some cheeses, is associated with favorable cardiometabolic outcomes, including inverse associations with incident cardiovascular disease, hypertension, and T2D risk [2,3,6]. Results from clinical trials have also shown neutral or favorable effects of dairy foods and beverages on metabolic risk factors, such as insulin sensitivity [12,[18], [19], [20]]. However, most studies have included both fermented and nonfermented dairy foods and did not differentiate by fat content, thus limiting the ability to draw inferences regarding subtypes of dairy foods on metabolic biomarkers.

To address whether the favorable metabolic outcomes from observational studies can be attributed to dairy fat or fermentation, this study was designed to assess the effects of including 2 servings/d of either *1*) full-fat fermented dairy foods; *2*) low-fat fermented dairy foods; or *3*) control foods with a similar macronutrient composition to the low-fat dairy foods on biomarkers of cardiometabolic health. We hypothesized that full-fat fermented dairy foods may have favorable effects on insulin sensitivity, the primary outcome variable, compared with low-fat fermented dairy and control foods because of its content of unique dairy fats. Individuals at risk of adverse cardiometabolic outcomes because of the presence of overweight or obesity and elevated fasting triglycerides (TGs) were recruited to participate in this study.

## Methods

### Study participants

Adults aged 18 to 74 y, with BMI between 25 and 39.9 kg/m^2^ and elevated fasting TG (≥135 mg/dL from September 2021 to September 2023 and ≥120 mg/dL from September 2023 through October 2024) were recruited for participation at 1 of the 6 testing sites (Supplemental Table 1). The original elevated TG inclusion criterion was based on prior estimates of TG in the top two-thirds of the population [21,22], but during trial recruitment, updated information was released on the distribution of fasting TG levels in the United States. Age-adjusted estimates of geometric mean TG concentrations among United States adults decreased from 113 mg/dL in 2007–2008 to 91 mg/dL in 2017–2018 [23]. A fasting TG of ≥120 mg/dL was set as the updated lower limit of the TG inclusion criterion because this concentration is ∼0.54 SDs, or 29 mg/dL, above the updated population geometric mean of 91 mg/dL in the United States [23], reflecting approximately the same fraction of the population that was originally estimated based on prior data. Additional inclusion criteria were adequate vein access, general good health based on history and screening laboratory tests, and willingness to maintain usual lifestyle habits and comply with study instructions. Exclusion criteria included fasting blood glucose ≥126 mg/dL or diagnosed type 1 diabetes or T2D, clinical signs of atherosclerotic cardiovascular disease, uncontrolled hypertension, unstable use of medications impacting lipid or carbohydrate metabolism, antibiotic use within 5 d of a clinic visit, recent weight change (±4.5 kg over 3 mo), major chronic diseases or infections, extreme dietary practices or allergies to the test foods, or a history of substance abuse or eating disorders. Females with childbearing age were required to use a medically approved form of contraception throughout the trial to prevent pregnancy during the study period. A urine pregnancy test was conducted for females under 60 y of age at screening and positive results were exclusionary.

### Study design

This was a randomized, controlled, parallel-arm, 12-wk trial with interim measurements at weeks 4 and 8. Participants meeting all the inclusion and none of the exclusion criteria at baseline were randomly assigned, and assignments were selected sequentially using sealed envelopes. Because of the use of various types of dairy foods and nondairy control foods, it was not possible to blind participants nor investigators to the treatment assignment. The study was conducted according to Good Clinical Practice Guidelines, the Declaration of Helsinki [24], and United States 21 Code of Federal Regulations [25]. The protocol was approved by the WCG IRB. Signed, written informed consent for participation in the study was obtained before conducting any protocol-specific procedures, and participants were informed of their right to withdraw from the study at any time.

### Study procedure

At screening, participants completed informed consent procedures, a medical history review, and underwent fasting measurements of blood glucose and blood lipid profiles to confirm eligibility. Eligible participants returned 1 wk later for baseline assessments, including a 3-d diet record assessment, anthropometrics, fasting blood draws, and a liquid meal tolerance test (LMTT). The LMTT included measurement of blood glucose and insulin before consuming 16 oz of Ensure (Abbott Laboratories) and at 30, 60, and 120 min after the start of consumption. Participants were then randomly assigned to 1 of 3 treatment groups using a computer-generated randomization scheme with a block size of 3: full-fat fermented dairy, low-fat fermented dairy, or a nondairy control group. A staff member selected the next sequential, sealed randomization envelope containing the randomization number and treatment for each participant. The randomization number, treatment, and code were maintained in each participant’s source documents. Study foods were provided with instructions on how to include the foods within the habitual diet with a focus on the incorporation of the study foods to maintain habitual energy intake.

**FIGURE 1:**
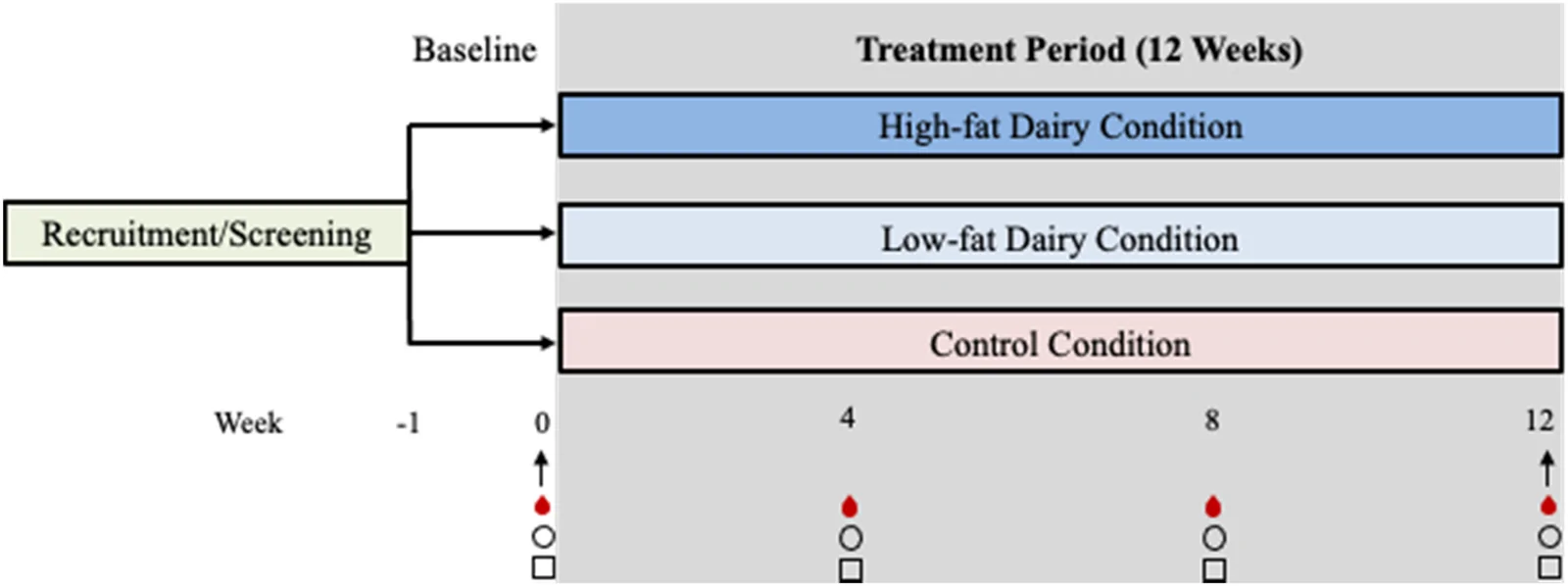
Study design. Liquid meal tolerance test assessment; Fasting blood draws for glucose, insulin, lipoprotein lipids, erythrocyte fatty acid profile, low density lipoprotein subfractions (baseline and week 12 only) and high-sensitivity C-reactive protein (baseline and week 12 only); Waist circumference and seated, resting systolic and diastolic blood pressures assessments; 3-d food diary. All end-of-diet condition testing was done in the follicular phase of the menstrual cycle of premenopausal women.

During the 12-wk intervention, participants returned to the clinic at weeks 4, 8, and 12 (Figure 1). Test visits were scheduled during the follicular phase of the menstrual cycle, when relevant. At each visit, compliance with study instructions was assessed with a daily food log and the amount of returned study food. Three-day diet records were collected and physical activity was assessed using the Stanford 7-day Physical Activity Recall Questionnaire [26]. Vital signs (resting heart rate and blood pressure) and anthropometric measurements (body weight and waist circumference) were recorded. Fasting blood samples were collected for primary and secondary outcomes. At week 12, a second LMTT was administered to evaluate postprandial glucose and insulin responses. Adverse events were assessed at each study visit with open-ended questions.

### Study products

Two servings per day of study foods were provided to participants every 2 or 4 wk and according to their randomization group with instructions on how to include the foods within the habitual diet. Participants chose when to consume their study foods each day. In the full-fat fermented dairy group, participants consumed 1 serving/d of full-fat fermented yogurt (8 fluid oz of flavored varieties) and 1 serving/d of full-fat fermented cheese (equivalent to 1.5 oz). Participants in the low-fat fermented dairy group consumed 1 serving/d of low-fat fermented yogurt (flavored varieties) and 1 serving/d of low-fat fermented cheese. The control group was provided with 2 options of nondairy, nonfermented foods. The first option consisted of 2 mini protein bars, and the second option consisted of 1 seasoned tuna pack, 4 crackers, and 1 granola bar. Study participants were provided with food options within each treatment group to enhance compliance and mimic a free-living setting. The daily energy and macronutrient contents of the low-fat fermented dairy group study products were matched with those of the control food study product options such that participants assigned to either of these treatment arms were consuming isocaloric, macronutrient-matched study foods. However, only daily servings and product type (fermented yogurt and fermented cheese) were matched between the full-fat fermented dairy and low-fat fermented dairy study products. Therefore, the daily nutrient and calorie levels for the full-fat fermented dairy study products were not matched to the low-fat fermented dairy and control study products to assess whether higher saturated fat content and energy density obtained from full-fat fermented dairy foods affect total daily nutrient intakes and cardiometabolic variables. The total daily macronutrient profiles of the study foods in each intervention group are provided in Table 1. Participants in all groups were allowed to consume ≤1 serving/d of 2% reduced-fat fluid cow's milk but otherwise were instructed to avoid additional dairy products. Participants were counseled on types of foods considered dairy foods (e.g., pizza with cheese, macaroni and cheese, cottage cheese, cream cheese). Subjects were instructed to otherwise maintain their usual habits, including physical activity and other lifestyle habits.

**TABLE 1:** Daily servings of intervention foods and their total daily macronutrient composition

Study foods/d	Full-fat fermented dairy foods	Low-fat fermented dairy foods	Control foods Option 1	Control foods Option 2
	8 oz full-fat yogurt (Whole Foods 365, Stonyfield) 1.5 oz cheese stick/round (Sargento, Bel Brands USA)	8 oz low-fat yogurt (Whole Foods 365, Stonyfield) 1.5 oz light cheese stick/round (Sargento, Bel Brands USA)	2 mini protein bars (Clif Bar & Company)	Seasoned tuna pack (StarKist) 4 crackers (Ferrero NA) Granola bar (Quaker)
Total daily macronutrient composition				
Energy (kcal)	340–380	230–267	240–280	298
Total fat (g)	19–21	7–11	7–10	9
Sat fat (g)	12–14	4–6.5	5–6	4
Cholesterol (mg)	60–67	30–43	0	30
Protein (g)	16–18	20	16–20	18
Total CHO (g)	25–29	24–27	28–30	35
Sugars (g)	19–24	18–21	18	14

Abbreviations: CHO, carbohydrate; Sat, saturated.

### Primary and secondary outcome variables

The primary outcome variable was the percentage change from baseline (week 0) to the end of treatment (week 12) in the Matsuda index of insulin sensitivity (MISI) [27]. MISI is calculated from the LMTT and is a validated index of whole-body insulin sensitivity against both the euglycemic clamp and the intravenous glucose tolerance test with minimal model analysis [[28], [29], [30]]. It was chosen to assess insulin sensitivity because, unlike the HOMA-IR, which only assesses a steady-state measure of insulin sensitivity in the fasting state, MISI is a dynamic measure of insulin sensitivity reflecting both hepatic and peripheral responsiveness to insulin in fasting and postprandial states [28,29]. Secondary outcomes included the change or percentage change from baseline to end of treatment for *1*) carbohydrate metabolism markers [fasting glucose and insulin, LMTT disposition index, HOMA-IR, and homeostatic model assessment for β-cell function (HOMA-B)] [30,31]; *2*) lipid metabolism markers [total cholesterol (TC), LDL cholesterol, HDL cholesterol, non-HDL cholesterol, VLDL cholesterol, TG, oxidized LDL, and small dense LDL cholesterol]; *3*) high-sensitivity C-reactive protein (hs-CRP); *4*) erythrocyte C15:0 (pentadecanoic acid), C17:0 (margaric acid), and *trans*-C16:1n*–*7 (*trans*-palmitoleic acid) content; *5*) waist circumference; and *6*) vital signs (resting systolic and diastolic blood pressure and heart rate). Calculations for MISI, HOMA-IR, HOMA-B, and disposition index can be found in the Supplemental Materials. Changes from baseline to 4 and 8 wk, when assessed, were considered exploratory. Baseline is the mean of the screening and week 0 values for fasting glucose and insulin, hs-CRP, lipids, and fatty acids. For all remaining outcomes, baseline is the week 0 value. End of treatment for all outcomes is the week 12 value.

### Laboratory measures for outcome variables

Fasting glucose and blood lipids at screening were collected and assessed via fingerstick using Cholestech LDX Lipid Glu Test Cassettes (Abbott Laboratories). Participants with fingerstick TG values ≥108 mg/dL provided fasting venous blood samples and were allowed to continue if the venous sample showed a value ≥120 mg/dL. A comprehensive chemistry profile, including sodium, potassium, carbon dioxide, glucose, blood urea nitrogen, creatinine, blood urea nitrogen/creatinine, calcium, total protein, albumin, globulin, total bilirubin, aspartate aminotransferase, and alanine aminotransferase, was also conducted at screening to evaluate general health and eligibility. Fasting blood samples were collected at baseline and during weeks 4, 8, and 12 to assess glucose, insulin, blood lipids, erythrocyte fatty acids, and hs-CRP. Lipoprotein subfractions were assessed at baseline and week 12 only.

An enzymatic method using cholesterol esterase and cholesterol oxidase conversion, followed by the Trinder endpoint, was used to determine TC using the Roche Integra 400 plus analyzer (Roche Diagnostics). HDL cholesterol was analyzed using accelerator select detergent methodology, and TGs were determined using enzymatic colorimetric testing and analyzed with the Dimension Vista System (Siemens Medical Solutions USA). LDL cholesterol was calculated according to the Friedewald equation in individuals with TG <400 mg/dL and the Martin–Hopkins method in individuals with TG ≥400 mg/dL [32]. VLDL cholesterol was calculated by subtracting LDL cholesterol and HDL cholesterol from TC. Oxidized LDL and cholesterol carried by small dense LDL were determined using a sandwich ELISA (LS Bio Vector Laboratories Inc.).

Briefly, hs-CRP samples were analyzed with the Siemens Atellica CH Analyzer using a latex-enhanced immunoturbidimetric method following the manufacturer’s protocol (Siemens Medical Solutions USA, Inc.). The fatty acid content of unwashed, packed red blood cells was isolated from fasted blood samples and processed as previously described [33]. The percentages of erythrocyte fatty acid composition of pentadecanoic acid (C15:0), margaric acid (C17:0), and *trans*-palmitoleic acid (*trans*-C16:1n*–*7) were determined using gas chromatography with flame ionization detection at OmegaQuant Analytics. Gas chromatography was carried out using a GC-2010 Gas Chromatograph (Shimadzu Corporation) [33]. Fatty acids were identified by comparison with a standard mixture of fatty acids (GLC 941, NuCheck Prep). Fatty acid composition was expressed as a percentage of the total identified fatty acids. The mean quality control coefficients of variation were 12.1%, 7.53%, and 16.0% for pentadecanoic acid (C15:0), margaric acid (C17:0), and *trans*-palmitoleic acid (*trans*-C16:1n*–*7), respectively.

### Diet record collection and analysis

Because of the different energy and macronutrient contents of the full-fat fermented dairy food study products and study products in the low-fat fermented dairy and control groups, dietary records were analyzed to determine daily energy, macronutrient, and key dairy micronutrient intakes in participants. Three-day diet records were provided and collected at baseline and during weeks 4, 8, and 12. The 3-d diet records were analyzed by using Trustwell Food Processor Nutrition Analysis software (version 11.11.32, Trustwell) and were used to assess total daily dietary nutrient intakes, which included nutrient intakes from the assigned study products.

### Waist circumference, heart rate, and blood pressure measurements

Waist circumference is one measure of adiposity and is strongly linked to higher energy and fat intakes, so waist circumference was assessed by taking 2 independent measurements, in centimeters, at the midway point between the top of the hip bone and the bottom of the lowest rib bone using a flexible tape measure. If the measurements differed by ≤0.5 cm, they were averaged, and the result was recorded as the waist circumference measurement. If the measurements differed by >0.5 cm, a third measurement was taken. Of the 3 measurements, the 2 that were closest were averaged and recorded. Heart rate and blood pressure were measured after the subject had been seated quietly for 5 min. An automated device (Welch Allyn or GE Dinamap V100, General Electric) took 3 measurements, each 1 min apart. The measurements taken at each time point were averaged and recorded for heart rate and blood pressures.

### Statistical analysis

A sample size of 42 participants per group was estimated a priori to provide 80% power to detect a 15% difference between groups (0.68 SD, assuming a pooled SD of 22%) for percent change from baseline in MISI, based on an α = 0.05, 2-sided [20,27,34]. Sample size calculations were performed for analysis of variance with 3 groups using the Primer of Biostatistics software package, version 6.0 (McGraw-Hill Medical). A total of 46 participants per group (138 total participants) were targeted to allow for attrition. Because of time and financial constraints resulting from the COVID-19 pandemic, recruitment was terminated at the end of 2024. Using the original assumptions for variability, the number of participants actually randomly assigned (*N* = 110) provided 73% power for detecting a 15% difference between groups.

The a priori statistical plan followed an intention-to-treat (ITT) approach, which included all randomly assigned participants. A secondary per protocol (PP) analysis included participants who completed the study with ≥80% compliance to study product intake and no major protocol deviations. Continuous variables were summarized as the mean and SD, geometric mean and back-transformed SD interval or SE, or the median and interquartile range limits, and categorical variables were summarized as frequencies and percentages. Descriptive statistics were examined to assess potential material differences among research sites in responses. Statistical analyses were conducted using R Statistical Software (v4.4.2; R Core Team, 2024). All statistical tests were tested at α = 0.05, 2-sided.

Differences between treatment groups in the percentage change in MISI from baseline to week 12 (primary outcome) were assessed using analysis of covariance models with treatment group as a fixed effect (full fat, low-fat, control) and baseline value as a covariate. When significant treatment effects were observed, differences in response between treatment groups were explored using Hochberg’s method to account for ≤3 comparisons. Normality of model residuals was evaluated visually and with quantile-quantile plots. Because several outcome variables showed departures from Gaussian normality, differences between groups in the primary outcome were assessed using models for the natural log of the ratio change from baseline, where ratio change was computed at the participant level as the value at week 12 divided by the baseline value. Least squares geometric mean ratios were converted to least squares geometric mean differences in percentage changes by subtracting the least squares geometric mean ratio from 1 and multiplying by 100. Because SEs and SDs are not symmetrical around geometric means, variability was expressed as the back-transformed mean ±1 SD of the natural log-transformed values, and the precision of least squares geometric means was expressed with 95% confidence intervals (CIs). Missing outcome data in the ITT population were imputed using the Markov Chain Monte Carlo method under the assumption that data were missing at random. Estimates were pooled across imputed datasets using Rubin’s Rules [35]. Treatment assignment and available outcome data were included in the imputation models.

For secondary outcomes assessed at baseline and week 12 only (disposition index, HOMA-IR, HOMA-B, apolipoprotein B, oxidized LDL, and small dense LDL cholesterol), differences between treatment groups in the percentage change from baseline to week 12 were assessed using the same modeling approach used for the primary outcome. For secondary outcomes assessed at baseline and weeks 4, 8, and 12 (lipids, hs-CRP, fatty acids, body composition, vitals, fasting glucose and insulin), differences between treatment groups in the percentage change from baseline to week 12 were assessed using repeated measures linear mixed models with treatment group (full fat, low-fat, control), study week (weeks 4, 8, 12), and treatment-by-week interaction as fixed effects, baseline value as a covariate, and participant as a random effect. An unstructured covariance structure was used to model the within-participant correlation across study weeks. When significant treatment or time effects, or treatment-by-time effects were observed, linear contrasts were performed to further explore differences. Percentage changes from baseline to weeks 4 and 8 were considered exploratory. For all secondary and exploratory outcomes, least squares geometric mean differences in percentage changes and their variability were computed from the least squares geometric mean ratios of the ratio changes, as described for the primary outcome. Cohen’s *d* was computed (Supplemental Material) for significant treatment effects. Missing outcome data in the ITT population were imputed using multiple imputation, as described for the primary outcome.

Given the established physiological relationship between visceral adiposity and metabolic risk, we assessed correlations between treatment-induced changes in waist circumference and changes in insulin sensitivity, markers of carbohydrate metabolism, and lipids to evaluate the biological consistency of our findings. Pearson correlations were generated separately for the natural log of the baseline values and the natural log of the ratio change from baseline to week 12 using observed values only. Dietary nutrient intake from each 3-d diet record was averaged, even if data for only 1 d were available. Potential differences between groups in daily dietary nutrient intake at week 12 were evaluated with the Kruskal–Wallis test. When significant treatment effects were observed, pairwise differences were explored using Dunn’s test with Hochberg method to account for ≤3 group comparisons. Potential differences in adverse events between groups were evaluated with chi-square tests.

## Results

**FIGURE 2:**
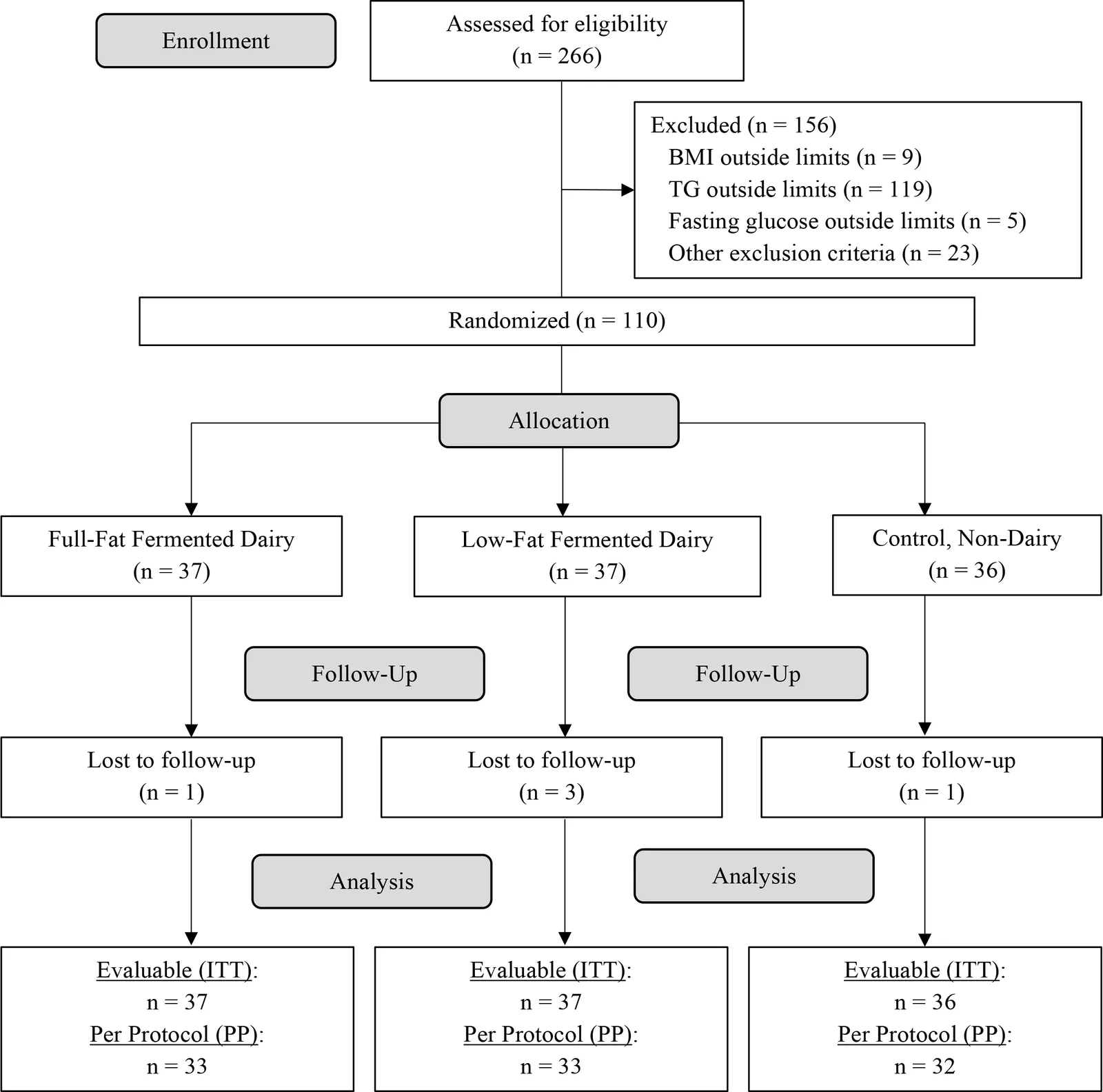
CONSORT diagram. ITT, intention-to-treat; TG, triglyceride.

Recruitment was terminated at the end of 2024. A total of 266 potential participants were screened and 110 were determined to be eligible and underwent randomization (Figure 2). Thirty-seven participants were randomly assigned to the full-fat and low-fat fermented dairy food groups each, and 36 participants were assigned to the nondairy control group. Therefore, there were 110 participants in the ITT population. The PP population consisted of 98 participants. Of the 110 randomly assigned participants, 5 withdrew from the trial before completion. The remaining 7 participants were not included in the PP population because of the following protocol violations: weight change of >±4% (*n =* 2); dairy food intake outside of study allowance during the study period (*n =* 1); nonapproved medication use because of sinus infection during the study period (*n =* 1); alcohol use within 24 h before test visit (*n =* 1); low compliance (11.6%) (*n =* 1); and alcohol use in excess of inclusion criteria allowance and antibiotic use during the study period (*n =* 1). The PP population baseline characteristics were not materially different from the ITT population (Supplemental Table 2), and no material differences in descriptive statistics were observed between research sites (data not shown).

Demographic and baseline characteristics of the participants randomly assigned to each intervention group can be found in Table 2. The overall mean (SD) age and BMI of the ITT population were 45.7 (13.5) y and 31.3 (4.28) kg/m^2^, respectively. Fifty-one (46.4%) participants were female. Ninety-one (82.7%) participants were White, 11 (10%) were Asian, 6 (5.5%) were Black/African American, and 2 (1.8%) indicated “Other” as their race; 55 (50.5%) participants were Hispanic/Latino and 54 (49.5%) were not Hispanic/Latino.

**TABLE 2:** Demographic and baseline characteristics of the intention-to-treat population (*N* = 110)^1^

	Full-fat	Low-fat	Control
	*n* = 37	*n* = 37	*n* = 36
Age^2^ (y)	41.4 (14.1)	47.9 (11.3)	47.9 (14.2)
Gender; *n* (%)			
Male	23 (62.2%)	20 (54.1%)	16 (44.4%)
Female	14 (37.8%)	17 (45.9%)	20 (55.6%)
Race; *n* (%)			
Asian,	6 (16.2%)	3 (8.1%)	2 (5.6%)
Black/African American	3 (8.1%)	3 (8.1%)	—
Other	—	1 (2.7%)	1 (2.8%)
White	28 (75.7%)	30 (81.1%)	33 (91.7%)
Ethnicity; *n* (%)			
Hispanic/Latino	18 (48.6%)	18 (48.6%)	19 (54.3%)
Not Hispanic/Latino	19 (51.4%)	19 (51.4%)	16 (45.7%)
Mean (SD)			
Height (cm)	168 (11.1)	166 (10.5)	166 (9.44)
Weight (kg)	86.5 (18.5)	88.3 (15.5)	86.1 (12.5)
BMI (kg/m^2^)	30.6 (4.40)	32.1 (3.99)	31.2 (4.42)
Waist circumference (cm)	100 (14.8)	102 (15.0)	102 (15.1)
Systolic blood pressure (mm Hg)	119 (8.68)	121 (9.41)	123 (10.8)
Diastolic blood pressure (mm Hg)	76.5 (6.92)	78.3 (7.72)	77.9 (7.27)
Heart rate (bpm)	73.5 (6.97)	72.3 (7.60)	72.0 (6.90)

Abbreviation: bpm, beats per minute.

1 Age, weight, waist circumference, BMI, blood pressures, and heart rate were assessed at baseline. All other data were assessed at the screening visit.

2 Years of age is provided as mean (SD).

Adverse event details are reported in Supplemental Table 3. Within the ITT population, the mean (SD) overall compliance with study product consumption was 98.4% (4.2%), 99.0% (2.9%), and 96.9% (9.2%) among the participants assigned to the full-fat fermented dairy, low-fat fermented dairy, and control food groups, respectively. Proportions of participants with 100% compliance with study product consumption throughout the entire study period were 77.8% in the full-fat dairy foods group, 82.9% in the low-fat dairy foods group, and 77.1% in the control foods group. The PP population had similar overall compliance compared with the ITT population.

Dietary intake data from 3-d diet records for the ITT group are shown in Table 3. There were statistically significant treatment group effects for intakes of total daily energy (*P* = 0.008), percentage of energy from carbohydrates (*P* = 0.005), grams of added sugar (*P* = 0.015), percentages of energy from total and saturated fats (*P* < 0.001 for both), milligrams of cholesterol (*P* = 0.014), and milligrams of calcium (*P* < 0.001). Percentage of energy from protein intake was not significantly different among the 3 treatment groups.

**TABLE 3:** Median (IQRL) energy and select nutrient intakes at baseline and after 12-wk intake of high-fat fermented dairy foods, low-fat fermented dairy foods, or control foods among the intention-to-treat population (*N* = 109)^1^

	Baseline, median (IQRL)	Week 12, median (IQRL)	*P* value^2^^,^^3^
Energy (kcal/d)			
Control	1596 (1455, 1937)	1633 (1391, 1969)	0.008^4^
Low-fat	1580 (1271, 1848)	1506 (1240, 1851)	
Full-fat	1691 (1465, 1981)	1916 (1628, 2175)	
Carbohydrates (% total energy)			
Control	50.2 (44.8, 55.4)	51.1 (44.0, 55.7)	0.005^5^
Low-fat	50.6 (47.8, 56.6)	47.1 (41.1, 53.0)	
Full-fat	51.2 (45.0, 58.6)	44.0 (38.5, 51.0)	
Sugars (% total energy)			
Control	14.5 (11.0, 20.2)	17.4 (12.9, 22.0)	0.184
Low-fat	16.5 (12.9, 21.5)	14.9 (11.8, 19.7)	
Full-fat	15.2 (10.4, 19.0)	14.0 (10.0, 19.0)	
Added sugars (g/d)			
Control	13.7 (8.13, 33.2)	22.9 (13.9, 52.7)	0.015^6^
Low-fat	17.5 (4.68, 35.5)	17.9 (13.0, 22.9)	
Full-fat	13.5 (7.29, 30.1)	23.7 (15.7, 36.3)	
Protein (% total energy)			
Control	16.8 (14.6, 21.0)	18.9 (16.0, 21.8)	0.086
Low-fat	17.1 (14.4, 20.1)	20.0 (17.9, 23.2)	
Full-fat	16.9 (14.4, 21.4)	17.1 (15.6, 22.2)	
Total fat (% total energy)			
Control	32.0 (25.6, 39.3)	30.1 (25.3, 34.7)	<0.001^7^
Low-fat	31.5 (28.0, 36.2)	30.9 (27.3, 39.1)	
Full-fat	31.3 (25.5, 36.1)	36.8 (33.5, 42.7)	
Saturated fat (% total energy)			
Control	8.62 (6.70, 11.9)	9.95 (8.12, 12.2)	<0.001^8^
Low-fat	8.56 (6.85, 11.5)	9.85 (8.26, 11.6)	
Full-fat	9.94 (7.32, 11.6)	13.8 (12.3, 15.0)	
Dietary fiber (g/d)			
Control	16.2 (11.3, 21.9)	13.8 (10.8, 19.7)	0.713
Low-fat	16.1 (11.9, 19.7)	12.9 (9.57, 16.8)	
Full-fat	13.9 (10.6, 18.1)	13.0 (9.45, 18.0)	
Cholesterol (mg/d)			
Control	284 (184, 381)	242 (191, 343)	0.014^9^
Low-fat	226 (148, 352)	200 (151, 311)	
Full-fat	320 (198, 430)	314 (207, 545)	
Calcium (mg/d)			
Control	326 (208, 565)	349 (192, 445)	<0.001^10^
Low-fat	349 (208, 523)	515 (437, 654)	
Full-fat	415 (246, 552)	706 (591, 831)	
Magnesium (mg/d)			
Control	103 (81.2, 161)	80.0 (56.3, 132)	0.239
Low-fat	103 (57.5, 140)	85.3 (47.3, 134)	
Full-fat	97.0 (70.0, 147)	105 (65.4, 171)	
Potassium (mg/d)			
Control	1820 (1433, 2156)	1349 (1072, 1896)	0.447
Low-fat	1393 (1089, 1895)	1347 (862, 1667)	
Full-fat	1510 (1207, 1943)	1447 (972, 2084)	
Sodium (mg/d)			
Control	2614 (2011, 3276)	2541 (2166, 3294)	0.677
Low-fat	2526 (1907, 3101)	2325 (1918, 2974)	
Full-fat	2612 (2153, 3256)	2552 (2140, 3217)	
Vitamin A (IU/d)			
Control	597 (279, 1328)	545 (369, 1598)	0.473
Low-fat	863 (200, 1353)	474 (40.2, 1290)	
Full-fat	592 (288, 1180)	367 (95.1, 1212)	
Vitamin D (IU/d)			
Control	54.8 (17.4, 153)	40.1 (6.75, 110)	0.696
Low-fat	36.4 (7.20, 85.7)	30.3 (6.35, 66.5)	
Full-fat	56.6 (19.7, 101)	49.9 (5.50, 93.8)	

Abbreviations: ANOVA, analysis of variance; IQRL, interquartile range limit.

1 Data are from 3-d diet records [Food Processor Nutrition Analysis & Fitness Software (Version 1.4)], and nutrients were averaged across the 3 days (or the number of available days).

2 *P* values are for treatment effect at week 12. *P* values were obtained from Kruskal–Wallis test (ANOVA model on ranked values) to test for differences between 3 independent treatment groups. *P* values are nominal and are not adjusted for multiple comparisons.

3 When a significant (*P* < 0.05) treatment effect at week 12 was observed, pairwise group differences were assessed using Dunn’s test with the Hochberg method to account for 3 group comparisons.

4 *P =* 0.007 for treatment effect of low-fat compared with full-fat.

5 *P =* 0005 for treatment effect of control compared with full-fat.

6 *P =* 0.045 for treatment effect of control compared with low-fat, and *P =* 0.024 for treatment effect of low-fat compared with full-fat.

7 *P* < 0.001 for treatment effect of control compared with full-fat, and *P =* 0.008 for treatment effect of low-fat compared with full-fat.

8 *P* < 0.001 for treatment effect of control compared with full-fat and low-fat compared with full-fat.

9 *P =* 0.012 for treatment effect of low-fat compared with full-fat.

10 *P* < 0.001 for treatment effect of control compared with full-fat, *P =* 0.001 for treatment effect of control compared with low-fat, and *P* = 0.002 for treatment effect of low-fat compared with full-fat.

**FIGURE 3:**
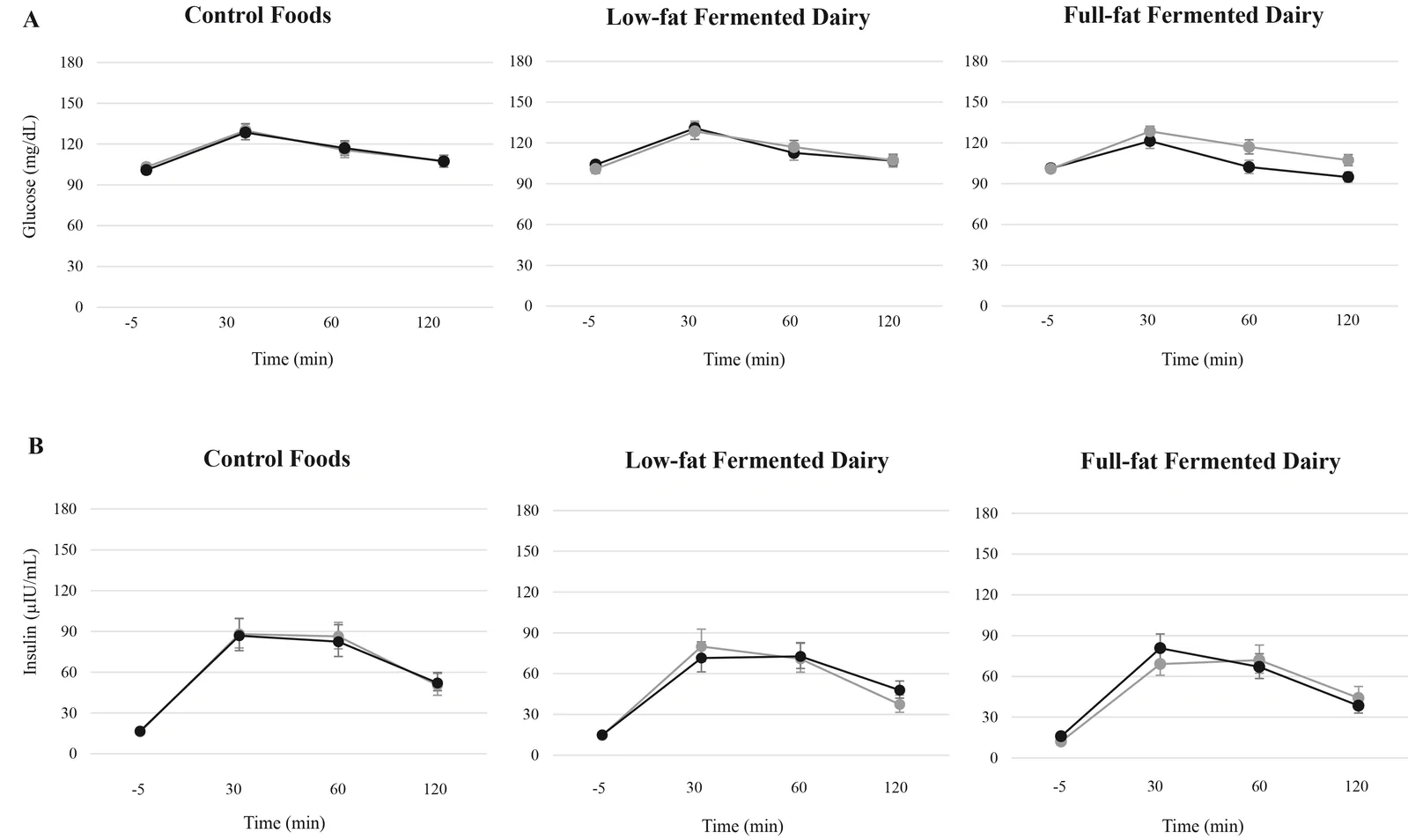
Geometric mean (SE) of glucose (A) and insulin (B) response to the liquid meal tolerance test at baseline (grey circles) and after 12-wk intervention (black circles) after control foods, low-fat fermented dairy foods, or full-fat fermented dairy foods among the intention-to-treat population (*N* = 110).

No differences across groups were observed for the primary outcome variable, MISI (Table 4) in the ITT population. The effect estimates for the difference (*P* > 0.05) in percent change in least squares geometric mean (95% CI) were 5.24% (−20.0, 38.5) for low-fat fermented dairy compared with control and −3.11% (−26.3, 27.3) for full-fat fermented dairy compared with control. The treatments also did not significantly affect any of the secondary outcome variables of carbohydrate metabolism, including disposition index, HOMA-IR, and HOMA-B (Table 4). Neither fasting glucose nor fasting insulin was significantly affected by any of the interventions (Table 4), and no effect was observed on glucose and insulin parameters during the LMTTs for any of the treatment groups (Figure 3). Similar results were obtained for the PP analyses (data not shown).

**TABLE 4:** Insulin sensitivity outcomes assessments at baseline and 12 wk after intake of high-fat fermented dairy foods, low-fat fermented dairy foods, or control foods among the intention-to-treat population (*N* = 110)^1^^,^^2^

	Baseline GM (−SD,+SD)	Week 12 GM (−SD,+SD)	% Δ from baseline LSGM (95% CI)	Difference in % Δ vs. control LSGM (95% CI)	Difference in % Δ full-fat vs. low-fat LSGM (95% CI)	*P* value^3^
MISI						
Control	2.93 (1.54, 5.54)	2.90 (1.46, 5.76)	−5.39 (−22.0, 14.7)			
Low-fat	3.36 (1.46, 7.71)	3.26 (1.63, 6.49)	−0.44 (−17.7, 20.5)	5.24 (−20.0, 38.5)		
Full-fat	3.76 (1.71, 8.26)	3.38 (1.62, 7.08)	−8.33 (−24.4, 11.2)	−3.11 (−26.3, 27.3)	−7.93 (−30.0, 21.1)	0.830
Disposition Index						
Control	1.88 (1.11, 3.17)	1.83 (1.10, 3.07)	−2.00 (−17.9, 17.0)			
Low-fat	1.80 (1.00, 3.24)	1.80 (0.98, 3.32)	−0.95 (−17.0, 18.2)	1.07 (−21.3, 29.7)		
Full-fat	2.15 (1.33, 3.47)	2.01 (1.03, 3.93)	−1.63 (−17.7, 17.5)	0.38 (−22.1, 29.2)	−0.69 (−22.8, 27.7)	0.996
HOMA-IR						
Control	4.08 (2.03, 8.19)	4.14 (1.96, 8.75)	0.99 (−21.6, 30.1)			
Low-fat	3.83 (1.57, 9.38)	3.84 (1.83, 8.06)	5.41 (−18.1, 35.7)	4.38 (−27.1, 49.4)		
Full-fat	3.02 (1.35, 6.77)	3.98 (1.42, 11.1)	23.9 (−4.04, 59.9)	22.7 (−14.5, 76.0)	17.5 (−18.0, 68.3)	0.497
HOMA-B						
Control	8.55 (3.82, 19.2)	10.1 (3.06, 33.1)	15.1 (−13.5, 53.2)			
Low-fat	7.05 (3.19, 15.6)	7.69 (4.28, 13.8)	7.85 (−18.9, 43.4)	−6.32 (−37.6, 40.7)		
Full-fat	6.46 (2.93, 14.3)	8.14 (3.43, 19.3)	42.8 (6.97, 90.5)	24.0 (−17.5, 86.4)	32.4 (−11.6, 98.3)	0.357
Fasting Glucose (mg/dL)						
Control	103 (91.2, 117)	101 (84.6, 120)	−2.51 (−6.86. 2.05)			
Low-fat	107 (91.1, 125)	104 (87.5, 124)	−1.93 (−6.27, 2.60)	0.51 (−6.03, 7.51)		
Full-fat	102 (91.5, 114)	101 (84.7, 121)	−1.65 (−5.99, 2.89)	0.86 (−5.52, 7.67)	0.35 (−6.07, 7.20)	0.643
Fasting Insulin (μIU/mL)						
Control	17.2 (8.13, 36.5)	16.6 (7.88, 35.1)	0.80 (−18.7, 25.0)			
Low-fat	15.9 (7.88, 32.2)	15.0 (7.90, 28.3)	−11.7 (−28.6, 9.19)	−8.81 (−33.5, 25.1)		
Full-fat	13.8 (7.12, 26.7)	15.9 (6.07, 41.8)	11.9 (−9.55, 38.4)	9.71 (−19.7, 49.8)	20.3 (−12.4, 65.2)	0.717

Abbreviations: % Δ, percent change; ANCOVA, analysis of covariance; GM, geometric mean; HOMA-B, homeostatic model assessment for β-cell function; LSGM, least squares geometric mean; MISI, Matsuda index of insulin sensitivity.

1 For fasting glucose and fasting insulin, estimates were generated from repeated measures linear mixed models for the natural log (ln) of the ratio change from baseline to weeks 4, 8, and 12 that included treatment group, study week, and treatment-by-week interaction as fixed effects, baseline as a covariate, and participant as a random effect. For all other outcomes, estimates were generated from ANCOVA models for the ln-transformed ratio change from baseline to week 12 with treatment group as a fixed effect and baseline as a covariate. Ratio changes were computed as the weeks 4, 8, or 12 value, divided by the baseline value. LSGM and the LSGM differences for the percentage change from baseline are the back-transformed LS means and LS mean differences for the ln-transformed ratio changes re-expressed as percentage change.

2 Missing values were imputed using multiple imputation (Markov Chain Monte Carlo method using a single chain to create 10 imputations). Pooled estimates were computed using Rubin's Rules; *n* = 34 to 37 for full-fat dairy foods, *n* = 34 to 37 for low-fat dairy foods, and *n* = 35 to 36 for nondairy control foods.

3 *P* values are based on the ANCOVA F-test for the treatment effect.

No statistically significant differences across groups were observed for biomarkers of lipid metabolism, including fasting TC, LDL cholesterol, HDL cholesterol, non-HDL cholesterol, VLDL cholesterol, TG, apolipoprotein B, oxidized LDL, and small dense LDL cholesterol (Table 5). There was also no significant difference observed for hs-CRP.

**TABLE 5:** Fasting blood lipoprotein lipid, particle, and subfraction outcomes and hs-CRP outcomes at baseline and 12 wk after intake of high-fat fermented dairy foods, low-fat fermented dairy foods, or control foods among the intention-to-treat population (*N* = 110)^1^^,^^2^

	Baseline GM (−SD,+SD)	Week 12 GM (−SD,+SD)	% Δ from baseline LSGM (95% CI)	Difference in % Δ vs. control LSGM (95% CI)	Difference in % Δ full-fat vs. low-fat LSGM (95% CI)	*P* value^3^
Total cholesterol (mg/dL)						
Control	181 (141, 232)	179 (140, 229)	−1.51 (−6.06, 3.27)			
Low-fat	180 (144, 226)	174 (140, 215)	−4.78 (−9.12, −0.23)	−3.43 (−9.84, 3.44)		
Full-fat	186 (149, 231)	185 (147, 234)	−0.11 (−4.67, 4.66)	0.30 (−6.43, 7.51)	3.87 (−3.03, 11.3)	0.157
LDL cholesterol (mg/dL)						
Control	101 (71.3, 143)	98.3 (67.5, 143)	−3.27 (−9.91, 3.86)			
Low-fat	102 (74.1, 140)	98.1 (69.7, 138)	−4.88 (−11.3, 2.03)	−1.71 (−11.3, 8.96)		
Full-fat	107 (80.5, 143)	108 (81.1, 145)	1.94 (−4.97, 9.35)	4.02 (−6.02, 15.1)	5.83 (−4.46, 17.2)	0.474
HDL cholesterol (mg/dL)						
Control	43.9 (32.9, 58.4)	42.2 (31.1, 57.3)	−3.61 (−8.56, 1.61)			
Low-fat	44.0 (35.8, 54.0)	41.9 (34.3, 51.1)	−5.45 (−10.2, −0.39)	−1.95 (−9.07, 5.74)		
Full-fat	46.8 (34.8, 62.9)	45.7 (33.1, 63.2)	−1.71 (−6.71, 3.56)	1.24 (−6.18, 9.23)	3.24 (−4.26, 11.3)	0.634
Non-HDL cholesterol (mg/dL)						
Control	136 (103, 179)	135 (103, 179)	0.24 (−5.28, 6.07)			
Low-fat	135 (102, 178)	130 (99.3, 171)	−3.52 (−8.76, 2.02)	−4.07 (−11.6, 4.01)		
Full-fat	136 (106, 176)	137 (104, 180)	−0.32 (−5.73, 5.40)	−0.08 (−7.87, 8.37)	4.18 (−4.06, 13.1)	0.130
VLDL cholesterol (mg/dL)						
Control	30.3 (19.7, 46.7)	30.1 (17.6, 51.5)	1.27 (−9.84, 13.7)			
Low-fat	29.8 (19.8, 44.8)	28.1 (18.5, 42.7)	−7.46 (−17.5, 3.76)	−8.00 (−22.3, 8.90)		
Full-fat	26.4 (17.6, 39.7)	24.4 (13.8, 43.1)	−8.95 (−18.8, 2.12)	−10.5 (−24.3, 5.82)	−2.73 (−17.7, 15.0)	0.228
Triglycerides (mg/dL)						
Control	179 (118, 272)	178 (104, 307)	1.82 (−10.4, 15.6)			
Low-fat	175 (120, 255)	168 (112, 250)	−6.23 (−17.3, 6.28)	−7.04 (−22.3, 11.3)		
Full-fat	153 (105, 224)	142 (83.1, 242)	−9.25 (−20.0, 2.92)	−11.3 (−25.7, 5.88)	−4.62 (−20.2, 14.0)	0.205
Apolipoprotein B (mg/dL)						
Control	94.8 (71.4, 126)	96.8 (73.8, 127)	1.97 (−4.49, 8.86)			
Low-fat	94.3 (69.5, 128)	91.4 (70.9, 118)	−2.86 (−8.67, 3.31)	−4.74 (−13.2, 4.50)		
Full-fat	93.8 (71.7, 123)	96.3 (73.3, 126)	2.61 (−3.86, 9.52)	0.63 (−8.00, 10.1)	5.64 (−3.47, 15.6)	0.423
Oxidized LDL (ng/mL)						
Control	55.1 (43.9, 69.1)	58.4 (44.0, 77.3)	3.83 (−4.27, 12.6)			
Low-fat	57.9 (44.4, 75.7)	57.4 (46.2, 71.2)	−0.26 (−7.50, 7.55)	−3.94 (−14.1, 7.43)		
Full-fat	56.7 (44.9, 71.8)	56.6 (44.5, 72.1)	−0.52 (−8.10, 7.70)	−4.19 (−14.0, 6.74)	−0.26 (−11.0, 11.8)	0.696
Small dense LDL cholesterol (mg/dL)						
Control	21.8 (16.7, 28.6)	21.5 (16.6, 27.9)	−0.82 (−7.61, 6.47)			
Low-fat	21.4 (15.7, 29.2)	22.2 (17.3, 28.6)	4.03 (−2.49, 11.0)	4.89 (−5.15, 16.00)		
Full-fat	21.3 (17.5, 25.9)	21.7 (17.1, 27.5)	0.99 (−5.86, 8.33)	1.82 (−7.31, 11.85)	−2.93 (−11.9, 6.96)	0.615
hs-CRP (mg/L)						
Control	2.04 (0.82, 5.05)	2.01 (0.65, 6.22)	−4.12 (−28.6, 28.8)			
Low-fat	1.63 (0.53, 5.00)	1.96 (0.72, 5.40)	24.0 (−7.33, 65.9)	25.2 (−17.2, 89.5)		
Full-fat	1.72 (0.51, 5.85)	1.51 (0.45, 5.03)	−15.5 (−36.8, 13.1)	−8.93 (−40.2, 38.8)	−27.3 (−52.0, 10.3)	0.278

Abbreviations: % Δ, percent change; ANCOVA, analysis of covariance; GM, geometric mean; hs-CRP, high-sensitivity C-reactive protein; LSGM, least squares geometric mean.

1 For apolipoprotein B, oxidized LDL, and small dense LDL cholesterol, estimates were generated from ANCOVA models for the natural log (ln) of the ratio change from baseline to week 12 with treatment group as a fixed effect and baseline as a covariate. For all other outcomes, estimates were generated from a repeated measures linear mixed model for the ln-transformed ratio change from baseline to weeks 4, 8, and 12 that included treatment group, study week, and treatment-by-week interaction as fixed effects, baseline as a covariate, and participant as a random effect. Ratio changes were computed as the weeks 4, 8, or 12 value, divided by the baseline value. LSGMs and the LSGM differences for the percentage change from baseline are the back-transformed LS means and LS mean differences for the ln-transformed ratio changes re-expressed as percentage change.

2 Missing values were imputed using multiple imputation (Markov Chain Monte Carlo method using a single chain to create 10 imputations). Pooled estimates were computed using Rubin's Rules; *n* = 36 to 37 for full-fat dairy foods, *n* = 31 to 37 for low-fat dairy foods, and *n* = 32 to 36 for nondairy control foods.

3 *P* values are based on the ANCOVA F-test for the treatment effect.

Assessment of erythrocyte fatty acid content showed that there was a near-significant treatment group effect (*P* = 0.053) for erythrocyte *trans*-palmitoleic acid (*trans*-C16:1n*–*7) content (Table 6). The 12-wk full-fat fermented dairy food intervention resulted in a near-significant least squares geometric mean (95% CI) decrease in erythrocyte *trans*-palmitoleic acid (*trans*-C16:1n–7) content by 19.0% (3.38%, 32.0%) compared with the control group (nominal *P* = 0.02; *P* = 0.059 after adjustment for multiple comparisons). No significant differences were observed when comparing the percentage changes of the low-fat fermented dairy and control groups. Neither pentadecanoic acid (C15:0) nor margaric acid (C17:0) showed differential responses across dietary conditions after 12 wk of intervention. There was no statistically significant effect of product on erythrocyte *trans*-palmitoleic acid (*trans*-C16:1n*–*7) content in the PP population analysis.

**TABLE 6:** Erythrocyte fatty acid content of pentadecanoic acid (C15:0), margaric acid (C17:0), and *trans*-palmitoleic acid (*trans*-16:1n–7), as a percentage of total fatty acids, at baseline and 12 wk after intake of high-fat fermented dairy foods, low-fat fermented dairy foods, or control foods among the intention-to-treat population (*N* = 110)^1^^,^^2^

	Baseline GM (+SD, −SD)	Week 12 GM (+SD, −SD)	% Δ from baseline LSGM (95% CI)	Difference in % Δ vs. control LSGM (95% CI)	Difference in % Δ full-fat vs. low-fat LSGM (95% CI)	*P* value^3^
C15:0—Pentadecanoic acid (% total fatty acids)						
Control	0.13 (0.10, 0.17)	0.13 (0.10, 0.17)	−4.88 (−12.2, 3.11)			
Low-fat	0.12 (0.09, 0.17)	0.12 (0.09, 0.17)	−1.07 (−8.62, 7.11)	1.84 (−9.98, 15.2)		
Full-fat	0.13 (0.11, 0.16)	0.13 (0.10, 0.17)	2.78 (−5.06, 11.3)	5.03 (−6.94, 18.5)	3.13 (−8.38, 16.1)	0.974
C17:0—Margaric acid (% total fatty acids)						
Control	0.32 (0.26, 0.39)	0.30 (0.26, 0.35)	−4.06 (−7.66, −0.32)			
Low-fat	0.31 (0.25, 0.38)	0.30 (0.24, 0.39)	−1.54 (−5.18, 2.25)	3.01 (−2.89, 9.27)		
Full-fat	0.31 (0.27, 0.36)	0.31 (0.27, 0.36)	0.01 (−3.70, 3.85)	4.81 (−0.95, 10.9)	1.75 (−3.88, 7.71)	0.342
*trans-*C16:1n–7—*trans*-Palmitoleic acid (% total fatty acids)						
Control	0.30 (0.19, 0.49)	0.33 (0.22, 0.48)	11.1 (−1.54, 25.3)			
Low-fat	0.31 (0.19, 0.48)	0.32 (0.20, 0.50)	4.14 (−7.55, 17.3)	−3.23 (−19.5, 16.3)		
Full-fat	0.27 (0.19, 0.40)	0.24 (0.15, 0.40)	−10.7 (−20.8, 0.60)	−19.0 (−32.0, −3.38)^4^	−16.3 (−30.1, 0.27)	0.053

Abbreviations: % Δ, percent change; ANCOVA, analysis of covariance; GM, geometric mean; LSGM, least squares geometric mean.

1 Estimates were generated from a repeated measures linear mixed model for the natural log (ln) of the ratio change from baseline to weeks 4, 8, and 12 that included treatment group, study week, and treatment-by-week interaction as fixed effects, baseline as a covariate, and participant as a random effect. Ratio changes were computed as the weeks 4, 8, or 12 value, divided by the baseline value. LSGMs and the LSGM differences for the percentage change from baseline are the back-transformed LS means and LS mean differences for the ln-transformed ratio changes re-expressed as percentage change.

2 Missing values were imputed using multiple imputation (Markov Chain Monte Carlo method using a single chain to create 10 imputations). Pooled estimates were computed using Rubin's Rules; *n* = 36 to 37 for full-fat dairy foods, *n* = 34 to 37 for low-fat dairy foods, and *n* = 35 to 36 for nondairy control foods.

3 *P* values are based on the ANCOVA F-test for the treatment effect. When a significant (*P* < 0.05) treatment effect was observed, pairwise differences between products were assessed within each study week using the Hochberg method to account for 3 group comparisons.

4 Nominal *P* value = 0.020 and adjusted *P* value = 0.059 after correction for multiple comparisons for the difference between control compared with full-fat at week 12.

There was a statistically significant (*P* = 0.036) group effect for waist circumference at week 12 (Table 7). Waist circumference increased by 2.21% in the control group and, in comparison, was reduced by 1.62% in the full-fat dairy group [effect estimate of −4.13% (−7.33, −0.81); *d*_*adj*_ = −0.58; *P* = 0.047 for the pairwise comparison]. No significant effect was observed on waist circumference between full-fat fermented dairy and low-fat fermented dairy [effect estimate of −2.59% (−5.83, 0.76); *d*_*adj*_ = −0.36; *P* = 0.254 for the pairwise comparison]. Within the ITT population, waist circumference was significantly inversely (*P* < 0.05) correlated with MISI at baseline in the overall study sample, the low-fat fermented dairy group, and the high-fat fermented dairy group and with HOMA-IR in the overall study sample (Supplemental Table 4). Waist circumference was also significantly inversely (*P* < 0.05) correlated with HDL cholesterol at baseline in the overall sample and the low-fat fermented dairy group. For ratio changes from baseline, there were only significant correlations between ratio changes in waist circumference in small dense LDL cholesterol, and this was observed in the overall sample (*r* = −0.345, *P* < 0.001), control group (*r* = −0.417, *P* = 0.014), and full-fat fermented dairy group (*r* = −0.492, *P* = 0.002). Body weight, blood pressures, and heart rate changes did not differ significantly across groups (Table 7). Results for the PP analysis were generally similar to those for the ITT analysis. However, the *P* value for the difference in the change from baseline in waist circumference at week 12 between the full-fat dairy and control groups did not reach statistical significance (*P* = 0.252).

**TABLE 7:** Waist circumference, body weight, blood pressures, and heart rate assessments at baseline and 12 wk after intake of high-fat fermented dairy foods, low-fat fermented dairy foods, or control foods among the intention-to-treat population (*N* = 110)^1^^,^^2^

	Baseline GM (−SD,+SD)	Week 12 GM (−SD,+SD)	% Δ from baseline LSGM (95% CI)	Difference in % Δ vs. control LSGM (95% CI)	Difference in % Δ full-fat vs. low-fat LSGM (95% CI)	*P* value^3^
Waist circumference (cm)						
Control	100 (82.3, 122)	103 (93.8, 114)	2.21 (−0.04, 4.51)			
Low-fat	101 (86.6, 117)	102 (87.6, 118)	0.78 (−1.41, 3.01)	−1.58 (−4.87, 1.82)		
Full-fat	99.3 (85.7, 115)	98.1 (85.0, 113)	−1.62 (−3.75, 0.57)	−4.13 (−7.33, −0.81)^4^	−2.59 (−5.83, 0.76)	0.036
Body weight (kg)						
Control	85.3 (73.9, 98.4)	85.3 (73.9, 98.4)	0.10 (−0.75, 0.95)			
Low-fat	87.1 (73.0, 103)	86.6 (72.6, 104)	0.19 (−0.64, 1.03)	−0.07 (−2.00, 1.90)		
Full-fat	84.8 (69.4, 104)	84.4 (68.5, 104)	−0.47 (−1.30, 0.36)	−0.24 (−2.10, 1.65)	−0.17 (−2.08, 1.77)	0.793
Systolic blood pressure (mm Hg)						
Control	122 (112, 133)	122 (114, 130)	0.25 (−1.46, 1.99)			
Low-fat	121 (112, 130)	121 (112, 132)	0.80 (−0.89, 2.52)	0.37 (−2.07, 2.88)		
Full-fat	119 (110, 128)	120 (111, 129)	0.55 (−1.15, 2.27)	0.35 (−2.12, 2.89)	−0.02 (−2.44, 2.46)	0.667
Diastolic blood pressure (mm Hg)						
Control	77.5 (70.5, 85.2)	77.9 (71.1, 85.4)	0.42 (−1.60, 2.47)			
Low-fat	77.9 (70.3, 86.3)	79.3 (73.7, 85.4)	2.25 (0.23, 4.32)	1.62 (−1.30, 4.63)		
Full-fat	76.2 (69.6, 83.4)	76.4 (69.8, 83.5)	−0.07 (−2.05, 1.96)	−0.69 (−3.54, 2.24)	−2.28 (−5.06, 0.59)	0.245
Heart rate (bpm)						
Control	71.7 (65.0, 79.0)	72.0 (65.9, 78.6)	0.58 (−2.09, 3.31)			
Low-fat	71.9 (64.0, 80.7)	72.2 (64.1, 81.3)	0.84 (−1.79, 3.54)	0.26 (−3.55, 4.22)		
Full-fat	73.2 (66.2, 80.9)	73.9 (66.5, 82.1)	1.50 (−1.16, 4.22)	1.03 (−2.75, 4.96)	0.77 (−3.01, 4.71)	0.544

Abbreviations: % Δ, percent change; ANCOVA, analysis of covariance; bpm, beats per minute; GM, geometric mean; LSGM, least squares geometric mean.

1 Estimates were generated from a repeated measures linear mixed model for the natural log (ln) of the ratio change that included baseline as a covariate and terms for visit, product, and a visit-by-product interaction. Ratio changes were computed as the week 12 value divided by the baseline value. LSGMs and the LSGM differences for the percent change from baseline are the back-transformed LS means and LS mean differences for the ln-transformed ratio changes re-expressed as percent change.

2 Missing values were imputed using multiple imputation (Markov Chain Monte Carlo method using a single chain to create 10 imputations). Pooled estimates were computed using Rubin's Rules; *n* = 36 to 37 for full-fat dairy foods, *n* = 34 to 37 for low-fat dairy foods, and *n* = 35 to 36 for nondairy control foods.

3 *P* values are based on the ANCOVA F-test for the treatment effect. *P* values ≤0.05 for treatment effect were assessed using 2-sided *P* value for difference between products using Hochberg adjustment for multiple comparisons within each study week.

4 Nominal *P* value = 0.016 and adjusted *P* value = 0.047 after correction for multiple comparisons.

## Discussion

This trial in participants with overweight or obesity and elevated TGs demonstrated that 12-wk of intake of 2 servings/d of either full-fat or low-fat fermented dairy foods, compared with intake of nondairy control foods, had no statistically significant impact on insulin sensitivity or other indicators of carbohydrate metabolism. Consumption of 2 servings/d of full-fat fermented dairy foods was associated with a small reduction (i.e., −4.13%) in waist circumference, compared with control foods, without any significant effects on blood pressures; heart rate; lipoprotein lipids; oxidized LDL; cholesterol carried by small, dense LDL particles; or hs-CRP, a biomarker of chronic inflammation.

Although some prior research findings suggested that dairy foods may favorably impact insulin sensitivity [12,18,20], we did not observe any significant effects. This is consistent with the findings of a narrative review who identified 6 randomized clinical trials assessing the effect of dairy fat on cardiometabolic health and related health outcomes, including carbohydrate metabolism, and concluded that consuming full-fat dairy products, compared with their lower-fat counterparts, likely do not affect these parameters [[36], [37], [38]]. A 12-wk intervention study by Schmidt et al. [39] conducted in 67 adults with metabolic syndrome to assess the effect of 3.3 servings/d of either full-fat dairy, low-fat dairy, or a dairy-limited diet on glucose tolerance and related carbohydrate metabolism showed that glucose tolerance was not affected by treatment assignment; however, MISI slightly decreased in the 2 dairy groups. This discrepancy from other trials may stem from significant differences in baseline glycated hemoglobin (HbA1c) levels among the 3 treatment groups (5.4%, 5.8%, and 5.7% for dairy-limited, low-fat dairy, and full-fat dairy, respectively; *P* < 0.001). The limited-dairy control group had normal HbA1c levels, whereas the dairy groups showed levels indicative of prediabetes. Thus, the decreases in insulin sensitivity in those groups may be attributed to a pre-existing metabolic disadvantage. Consistent with our current findings, previous research from our laboratory also showed that 3 servings/d of dairy (reduced-fat milk and yogurt) had a neutral effect on insulin sensitivity and disposition index; however, sugar-sweetened product intake significantly worsened these parameters in otherwise healthy adults with obesity [15].

Within dairy products, fermentation, regardless of fat content, may have unique impacts on carbohydrate metabolism. Among fermented dairy foods, yogurt in particular has been shown to be favorably associated with indicators of carbohydrate metabolism and incidence of diabetes, prompting the Food and Drug Administration (FDA) to approve a qualified health claim in 2024 that states, “Eating yogurt regularly, ≥2 cups (3 servings) per week, may reduce risk of T2D according to limited scientific evidence” [40,41]. This claim is considered suitable to apply to all yogurts that meet the FDA’s standard of identity [42], including full-fat versions of yogurts. Because of this reason, both full-fat and low-fat fermented dairy foods were included in this study to assess whether higher fat content within the fermented dairy products affects cardiometabolic health variables compared with their lower-fat versions. The results of the current trial did not show differences in insulin sensitivity or other T2D risk factors between consumption of fermented dairy foods and control foods, regardless of whether full-fat or low-fat varieties were consumed. Notably, we observed an ∼4% to 6% difference between the full-fat and low-fat fermented dairy conditions for LDL cholesterol, non-HDL cholesterol, and apolipoprotein B after the 12-wk intervention. The study was underpowered to detect differences of these magnitudes, which may have potential clinical relevance over long periods, despite the lack of statistical significance.

There was a small but statistically significant reduction in waist circumference in participants consuming full-fat fermented dairy foods compared with those consuming control foods in the current trial. The reduced waist circumference was observed despite higher total energy intake in the full-fat fermented dairy treatment group compared with the low-fat fermented dairy group, and despite no significant changes in body weight across groups. Of note, the full-fat fermented dairy group reported significantly higher intake of energy than the low-fat dairy group. However, because there were no significant treatment effects on body weight, this may be an artifact of diet record reporting. Energy intake is generally underreported overall [43], but is more accurately reported for study foods, which may bias energy intake results higher for the full-fat fermented dairy food group. Schmidt et al. [39] found that high-fat dairy group participants gained a modest, but statistically significant, amount of weight (1.0 kg), compared with the dairy-limited control group.

A reduced waist circumference with intake of full-fat fermented dairy is consistent with findings from prior research suggesting that full-fat yogurt, compared with low-fat yogurt, is associated with reduced waist circumference [44], and yogurt intake, regardless of fat content, is linked to modestly favorable waist circumference changes compared with milk intake [45]. Moreover, there is some evidence from observational studies that full-fat dairy intake is associated with less body fat accumulation [46]. The potential impact of full-fat dairy on body composition warrants further investigation in larger and longer-term trials with more precise measures of changes in body composition. Additionally, although gut microbiome analysis was not included in this study, it is important to note that fermented dairy can affect the gut microbiome [47,48] and may be a plausible explanation for the observed waist circumference reduction, because gut microbiome composition has been linked to changes in waist circumference [49]. Intervention trials are also needed to determine whether dairy fat concentration affects potential microbiome-mediated mechanisms on waist circumference. Caution is warranted regarding the interpretation of this study’s finding because this was one of several secondary outcome variables, so the possibility of a type 1 statistical error exists. However, our results do not support the view that intake of full-fat dairy is likely to lead to weight gain compared with low-fat dairy.

Results from prior research indicate that intake of full-fat dairy does not adversely affect biomarkers of lipoprotein lipid metabolism and related variables and, in some instances, full-fat dairy has favorable effects. The authors of a meta-analysis of 19 randomized controlled trials with 1427 participants found that high dairy intake, regardless of fat content, was not associated with changes in levels of cholesterol carried by apo B-containing lipoproteins [45], and although Taormina et al. [50] found that full-fat yogurt significantly reduced circulating TG levels compared with nonfat yogurt, other lipoprotein lipid markers did not differ between the 2 treatments. Results from our prior trial also indicated that dairy had a neutral effect on lipoprotein lipid metabolism compared with nondairy. A randomized crossover trial found that replacing low-fat dairy with full-fat dairy in the Dietary Approaches to Stop Hypertension (DASH) diet for 3 wk significantly lowered TG and VLDL particle concentrations, while increasing LDL peak particle diameter, potentially reducing atherogenicity [51]. Additionally, a small trial assessing 2-wk of intake of 4.4 oz/d of cheese on lipoprotein lipid metabolism found no effect [52], nor did increasing intake of a variety of full-fat milk, yogurt, and cheese from 1.9 to 3.5 servings/d for 12 mo [53]. Our results are consistent with those from prior trials in that neither low-fat nor full-fat fermented dairy products had material effects on lipoprotein-related biomarkers compared with control products.

We did not observe any effect of dairy intake on blood pressures in this trial. However, the DASH study authors and those of several meta-analyses on dietary patterns and blood pressures have consistently concluded that dairy foods play a role in reducing blood pressures in both hypertensive and normotensive individuals [[54], [55], [56]], and this effect does not appear to depend on fat concentration. One plausible explanation for our observed lack of effect may be that, although some research has shown that dairy food intake can lower blood pressure in normotensive individuals (with mean baseline systolic/diastolic pressures ∼120/80 mm Hg for study participants), most trials have been conducted in hypertensive individuals. Any dairy-mediated blood pressure effects in normotensive individuals have been less pronounced than those observed in their hypertensive counterparts [57]. Consistent with our findings, a trial conducted by our research team comparing the intake of 3 servings/d of low-fat dairy foods with that of 3 servings/d of nondairy foods for 5 wk also found no significant effect of dairy intake on blood pressures; however, subgroup analysis indicated that the dairy intervention improved a measure of endothelial dilation response in those with compromised endothelial function [58]. Prior clinical trials assessing intake of fermented dairy compared with nonfermented dairy on metabolic parameters, including blood pressures, showed that fermentation of dairy, per se, did not independently affect blood pressures, because all dairy intervention groups reduced systolic and diastolic pressures to a similar degree [59,60].

The results of this trial also indicate that fermented dairy intake, regardless of fat concentration, did not affect a key biomarker of inflammation, hs-CRP, which is consistent with findings from prior research [[61], [62], [63]]. Erythrocyte fatty acid composition of 3 main dairy fatty acids, pentadecanoic acid (C15:0), margaric acid (C17:0), and *trans*-palmitoleic acid (*trans*-C16:1n*–*7), was also assessed in this study, because these fatty acids are markers of dairy fat intake and have been linked to lower incidence of T2D [17,64]. Circulating *trans*-palmitoleic acid (*trans*-C16:1n–7) can be generally regarded as a biomarker of ruminant dairy fat intake in the United States with unique characteristics that may contribute to several beneficial cardiometabolic markers observed with dairy intake [65,66]. However, the results showed no statistically significant effect of fermented dairy food intake, regardless of fat concentration, on the erythrocyte fatty acid composition of these fatty acids. Of note, we observed a modest (∼11%) reduction of *trans*-palmitoleic acid (*trans*-C16:1n–7) with full-fat fermented dairy food intake compared with an increase of a similar magnitude in the control food group. There may be several reasons for this observation. First, factors such as the ripening process of cheese can affect the amount of *trans*-palmitoleic acid (*trans*-C16:1n–7) in the product [67], so the decrease observed in circulating *trans*-C16:1n–7 may be indicative of a decrease in ripened cheese intake during the study period compared with participants’ typical dietary intakes. Second, dairy fat intake in the present trial may not have reached the concentration necessary to alter erythrocyte content of these fatty acids over a 12-wk period. The trial provided ∼17 g/d of dairy fat for 12 wk in the full-fat fermented dairy food intervention, whereas an intervention in a prior trial assessing *trans*-palmitoleic acid (*trans*-C16:1n–7) as a biomarker for dairy fat intake provided 28.5 g/d dairy fat for 12 wk [68]. An alternative hypothesis is that the reduced circulating levels may be indicative of the dairy fat’s relocation to tissue-specific sites, for example, abdominal adipose tissue, potentially propagating an alteration in visceral fat content and ultimately, reduced waist circumference. However, this is speculative, and studies are needed to confirm or refute this hypothesis.

This study has several strengths and limitations. A primary strength is that it was designed to reflect a “real-life” setting both in the study population and in the dietary intervention. Specifically, two-thirds of the United States population are overweight or have obesity, and many in these groups have elevated TGs or related metabolic disturbances. Additionally, the 8-fluid oz serving of full-fat or low-fat yogurt and the 1.5 oz serving of full-fat or light cheese are realistic fermented dairy foods and portion sizes that can be incorporated into a habitual diet. High compliance was reported in the fermented full-fat and low-fat dairy groups, supporting the feasibility of incorporating these levels of dairy foods in the habitual diet.

A limitation was the time and financial constraint resulting from the pandemic, causing enrollment to be terminated with 110 participants enrolled instead of the 126 participants (42 per treatment intervention) originally planned. As a result, the statistical power was reduced. Small differences in some secondary outcome variables may have reached statistical significance with a larger sample size. Furthermore, the possibility of type 1 statistical errors cannot be excluded, including the observed effect on waist circumference. Other limitations include the lack of study product blinding and no assessment of potential interactions with other foods or nutritional components that may account for the observed results. Furthermore, this trial did not have a control group matched for energy intake to that of the full-fat dairy condition, and the control intervention was a mixture of different food items and depended on the participants’ preferences. Although any variability related to offering such options is likely to be far smaller than variability in the background diet in free-living participants, the primary comparisons could theoretically be biased because the true unmeasured effects of the control intervention, including food displacement, may affect the findings.

In conclusion, 12 wk of intake of 2 daily servings of low-fat or full-fat fermented dairy foods, as compared with intake of nondairy control foods, did not significantly impact insulin sensitivity or other indicators of carbohydrate metabolism, biomarkers of lipoprotein metabolism, blood pressures, heart rate, or hs-CRP. There was a small but statistically significant reduction in waist circumference in the full-fat fermented dairy food group compared with the control group. These findings support the latest DGA recommendations that allow for the inclusion of full-fat dairy foods, as part of a healthful dietary pattern. Additional research is warranted to further investigate the impact of longer-term consumption of full-fat fermented dairy products on body composition and body fat distribution.

## Author contributions

The authors’ responsibilities were as follows – KCM, IE: designed the research; NG-M, IE, CGA, LAS, KLS, EJ, ANF, EG, BB-F: conducted the research; KCM, NG-M, IE, MLW, LAS, KLS, EJ, EG, BB-F: analyzed data and/or performed statistical analysis; OMP: prepared the manuscript; all authors: reviewed and edited the manuscript; KCM: had primary responsibility for the final content; and all authors: have read and approved the manuscript.

## Data availability

Data described in the manuscript, code book, and analytic code will be made available upon reasonable request to the corresponding author.

## Declaration of Generative AI and AI-assisted technologies in the writing process

The authors declare that no generative AI or AI-assisted technologies were used in the writing of this manuscript.

## Funding

This study was supported by the National Dairy Council. The funding sponsor provided comments on early aspects of the study design. The final decisions for all aspects of the study execution and the manuscript content were those of the authors alone.

## Conflict of interest

KCM reports research grants from ACH Food Companies, Cargill, General Mills, The Hershey Company, PepsiCo, Hass Avocado Board, National Cattlemen’s Beef Association, and National Dairy Council and consulting or advisory board positions with ACH Food Companies, Bragg Live Food Products, Campbell’s Company, National Cattlemen’s Beef Association, National Dairy Council, and Soy Nutrition Institute Global. IE and BB-F report research grants from Hass Avocado Board, National Mango Board, California Colors, and American Pecan Promotion Board. NG-M serves on the scientific advisory board for Haleon Wellness. ANF serves on the scientific advisory board for Eli Lilly and Company, Novo Nordisk, Gila Therapeutics, and Morphic Medical. All other authors report no conflicts of interest.

## References

[1] World Health Statistics 2025: Monitoring Health for the SDGs, Sustainable Development Goals. World Health Organization. [Internet]. 2025. Available from: https://iris.who.int/server/api/core/bitstreams/c992fbdc-11ef-43db-a478-7e7a195403ae/content.

[2] Zhuang P., Liu X., Li Y., Ao Y., Wu Y., Ye H. (2025). A global analysis of dairy consumption and incident cardiovascular disease. Nat. Commun..

[3] Drouin-Chartier J.P., Brassard D., Tessier-Grenier M., Côté J.A., Labonté M., Desroches S. (2016). Systematic review of the association between dairy product consumption and risk of cardiovascular-related clinical outcomes. Adv. Nutr..

[4] Gijsbers L., Ding E.L., Malik V.S., de Goede J., Geleijnse J.M., Soedamah-Muthu S.S. (2016). Consumption of dairy foods and diabetes incidence: a dose-response meta-analysis of observational studies. Am. J. Clin. Nutr..

[5] Hruby A., Ma J., Rogers G., Meigs J.B., Jacques P.F. (2017). Associations of dairy intake with incident prediabetes or diabetes in middle-aged adults vary by both dairy type and glycemic status. J. Nutr..

[6] Soedamah-Muthu S.S., de Goede J. (2018). Dairy consumption and cardiometabolic diseases: systematic review and updated meta-analyses of prospective cohort studies. Curr. Nutr. Rep..

[7] US Department of Agriculture and US Department of Health and Human Services, Dietary Guidelines for Americans, 2025–2030, tenth ed., US Department of Agriculture and US Department of Health and Human Services. [Internet]. 2025. Available from: https://cdn.realfood.gov/DGA.pdf.

[8] Fulgoni K., Fulgoni V.L., Agarwal S., Ricklefs-Johnson K., Pikosky M.A., Cifelli C.J. (2025). Current contribution to energy and nutrient intake from dairy foods in children and adults using NHANES, 2015–2018. J. Nutr..

[9] US Department of Agriculture and US Department of Health and Human Services, Dietary Guidelines for Americans, 2020-2025, ninth ed, US Department of Agriculture and US Department of Health and Human Services. [Internet]. 2020. Available from: https://www.dietaryguidelines.gov/sites/default/files/2020-12/Dietary_Guidelines_for_Americans_2020-2025.pdf.

[10] US Department of Agriculture and US Department of Health and Human Services, The Scientific Foundation for the Dietary Guidelines for Americans, 2025–2030, US Department of Agriculture and, US Department of Health and Human Services. [Internet]. 2025. Available from: https://cdn.realfood.gov/Scientific%20Report%20Appendices_FINAL_1.28.26.pdf.

[11] Drehmer M., Pereira M.A., Schmidt M.I., Alvim S., Lotufo P.A., Luft V.C. (2016). Total and full-fat, but not low-fat, dairy product intakes are inversely associated with metabolic syndrome in adults. J. Nutr..

[12] Mozaffarian D. (2016). Dietary and policy priorities for cardiovascular disease, diabetes, and obesity: a comprehensive review. Circulation.

[13] Pimpin L., Wu J.H., Haskelberg H., Del Gobbo L., Mozaffarian D. (2016). Is butter back? A systematic review and meta-analysis of butter consumption and risk of cardiovascular disease, diabetes, and total mortality. PLOS ONE.

[14] Guasch-Ferré M., Becerra-Tomás N., Ruiz-Canela M., Corella D., Schröder H., Estruch R. (2017). Total and subtypes of dietary fat intake and risk of type 2 diabetes mellitus in the Prevención con Dieta Mediterránea (PREDIMED) study. Am. J. Clin. Nutr..

[15] Liang J., Zhou Q., Kwame Amakye W., Su Y., Zhang Z. (2018). Biomarkers of dairy fat intake and risk of cardiovascular disease: a systematic review and meta analysis of prospective studies. Crit. Rev. Food Sci. Nutr..

[16] Yakoob M.Y., Shi P., Willett W.C., Rexrode K.M., Campos H., Orav E.J. (2016). Circulating biomarkers of dairy fat and risk of incident diabetes mellitus among men and women in the United States in two large prospective cohorts. Circulation.

[17] Imamura F., Fretts A., Marklund M., Ardisson Korat A.V., Yang W.S., Lankinen M. (2018). Fatty acid biomarkers of dairy fat consumption and incidence of type 2 diabetes: a pooled analysis of prospective cohort studies. PLoS Med.

[18] Maki K.C., Phillips A.K. (2015). Dietary substitutions for refined carbohydrate that show promise for reducing risk of type 2 diabetes in men and women. J. Nutr..

[19] Maki K.C., Nieman K.M., Schild A.L., Kaden V.N., Lawless A.L., Kelley K.M. (2015). Sugar-sweetened product consumption alters glucose homeostasis compared with dairy product consumption in men and women at risk of type 2 diabetes mellitus. J. Nutr..

[20] Turner K.M., Keogh J.B., Clifton P.M. (2015). Dairy consumption and insulin sensitivity: a systematic review of short- and long-term intervention studies. Nutr. Metab. Cardiovasc. Dis..

[21] Fan W., Philip S., Granowitz C., Toth P.P., Wong N.D. (2019). Hypertriglyceridemia in statin-treated US adults: the National Health and Nutrition Examination Survey. J. Clin. Lipidol..

[22] Ganda O.P., Bhatt D.L., Mason R.P., Miller M., Boden W.E. (2018). Unmet need for adjunctive dyslipidemia therapy in hypertriglyceridemia management. J. Am. Coll. Cardiol..

[23] Aggarwal R., Bhatt D.L., Rodriguez F., Yeh R.W., Wadhera R.K. (2022). Trends in lipid concentrations and lipid control among US adults, 2007-2018. JAMA.

[24] World Medical Association, Declaration of Helsinki: Ethical Principles for Medical Research Involving Human Subjects, 52nd WMA General Assembly, Edinburgh, Scotland. [Internet]. 2000. Available from: https://www.wma.net/what-we-do/medical-ethics/declaration-of-helsinki/doh-oct2000/.

[25] United States Food and Drug Administration, Title 21: Food and Drugs, United States Food and Drug Administration. [Internet]. 2025. Available from: https://www.ecfr.gov/current/title-21.

[26] Richardson M.T., Ainsworth B.E., Jacobs D.R., Leon A.S. (2001). Validation of the Stanford 7-day recall to assess habitual physical activity. Ann. Epidemiol..

[27] Matsuda M., DeFronzo R.A. (1999). Insulin sensitivity indices obtained from oral glucose tolerance testing: comparison with the euglycemic insulin clamp. Diabetes Care.

[28] R. Muniyappa, R. Madan, R.T. Varghese, Assessing Insulin Sensitivity and Resistance in Humans. Endotext. [Internet]. 2024. Available from: https://www.ncbi.nlm.nih.gov/books/NBK278954/.

[29] Moriyama K. (2025). Mini-review on insulin resistance assessment: advances in surrogate indices and clinical applications. World J. Clin. Cases..

[30] Maki K.C., Kelley K.M., Lawless A.L., Hubacher R.L., Schild A.L., Dicklin M.R. (2011). Validation of insulin sensitivity and secretion indices derived from the liquid meal tolerance test. Diabetes Technol. Ther.

[31] Santos-Báez L.S., Diaz-Rizzolo D.A., Borhan R., Popp C.J., Sordi-Guth A., DeBonis D. (2025). Predictive models of post-prandial glucose response in persons with prediabetes and early onset type 2 diabetes: a pilot study. Diabetes Obes. Metab..

[32] Martin S.S., Blaha M.J., Elshazly M.B., Toth P.P., Kwiterovich P.O., Blumenthal R.S. (2013). Comparison of a novel method vs the Friedewald equation for estimating low-density lipoprotein cholesterol levels from the standard lipid profile. JAMA.

[33] Harris W.S., Pottala J.V., Vasan R.S., Larson M.G., Robins S.J. (2012). Changes in erythrocyte membrane trans and marine fatty acids between 1999 and 2006 in older Americans. J. Nutr..

[34] Turner K.M., Keogh J.B., Clifton P.M. (2015). Red meat, dairy, and insulin sensitivity: a randomized crossover intervention study. Am. J. Clin. Nutr..

[35] RUBIN D.B. (1976). Inference and missing data. Biometrika.

[36] Taormina V.M., Unger A.L., Kraft J. (2024). Full-fat dairy products and cardiometabolic health outcomes: does the dairy-fat matrix matter?. Front. Nutr..

[37] Raziani F., Tholstrup T., Kristensen M.D., Svanegaard M.L., Ritz C., Astrup A. (2016). High intake of regular-fat cheese compared with reduced-fat cheese does not affect LDL cholesterol or risk markers of the metabolic syndrome: a randomized controlled trial. Am. J. Clin. Nutr..

[38] Engel S., Elhauge M., Tholstrup T. (2018). Effect of whole milk compared with skimmed milk on fasting blood lipids in healthy adults: a 3-week randomized crossover study. Eur. J. Clin. Nutr..

[39] Schmidt K.A., Cromer G., Burhans M.S., Kuzma J.N., Hagman D.K., Fernando I. (2021). The impact of diets rich in low-fat or∖ full-fat dairy on glucose tolerance and its determinants: a randomized controlled trial. Am. J. Clin. Nutr..

[40] United States Food and Drug Administration, Response letter to petition for a qualified health claim for yogurt and reduced risk of type 2 diabetes mellitus (Docket No. FDA-2019-P-1594) [Internet]. Available from: https://www.fda.gov/media/176608/download.

[41] Freitas M., O’Connor A., Blechman A., Cifelli C.J., Reinhardt Kapsak W. (2025). Yogurt and reduced risk of type 2 diabetes: exploring the Food and Drug Administration qualified health claim and potential implications for improving public health. J. Nutr..

[42] United States Food and Drug Administration, Yogurt standard of identity, Code of Federal Regulations, Title 21, Part 131.200 [Internet]. Available from: https://www.ecfr.gov/current/title-21/chapter-I/subchapter-B/part-131/subpart-B/section-131.200.

[43] Ravelli M.N., Schoeller D.A. (2020). Traditional self-reported dietary instruments are prone to inaccuracies and new approaches are needed. Front. Nutr..

[44] Santiago S., Sayón-Orea C., Babio N., Ruiz-Canela M., Martí A., Corella D. (2016). Yogurt consumption and abdominal obesity reversion in the PREDIMED study. Nutr. Metab. Cardiovasc. Dis..

[45] Kiesswetter E., Stadelmaier J., Petropoulou M., Morze J., Grummich K., Roux I. (2023). Effects of dairy intake on markers of cardiometabolic health in adults: a systematic review with network meta-analysis. Adv. Nutr..

[46] Zeitoun T., Chen Z.H., Burgner D., MacKechnie G., Huntington P., Mansell T. (2026). Milk fat intake, adiposity, and obesity in Canadian children: findings from the prospective Canadian CHILD cohort study. Am. J. Clin. Nutr..

[47] Aslam H., Marx W., Rocks T., Loughman A., Chandrasekaran V., Ruusunen A. (2020). The effects of dairy and dairy derivatives on the gut microbiota: a systematic literature review. Gut Microbes.

[48] Ní Chonnacháin C., Feeney E.L., Gollogly C., Shields D.C., Loscher C.E., Cotter P.D. (2024). The effects of dairy on the gut microbiome and symptoms in gastrointestinal disease cohorts: a systematic review. Gut Microbiome (Camb.)..

[49] Borrego-Ruiz A., Borrego J.J. (2025). The gut microbiome in human obesity: a comprehensive review. Biomedicines.

[50] Taormina V.M., Eisenhardt S., Gilbert M.P., Poynter M.E., Kien C.L., Kraft J. (2025). Full-fat yogurt compared with non-fat yogurt reduces blood triacylglycerol concentrations and lowers the triacylglycerol content in specific lipoprotein subclasses in adults with prediabetes: an exploratory analysis of a randomized-controlled trial. Lipids Health Dis.

[51] Chiu S., Bergeron N., Williams P.T., Bray G.A., Sutherland B., Krauss R.M. (2016). Comparison of the DASH (Dietary Approaches to Stop Hypertension) diet and a higher-fat DASH diet on blood pressure and lipids and lipoproteins: a randomized controlled trial. Am. J. Clin. Nutr..

[52] Høstmark A.T., Lunde M.S.H., Hjellset V.T. (2018). Short communication: daily intake of 125 g of cheese for 2 weeks did not alter amount or distribution of serum lipids or desaturase indexes in healthy adults in an exploratory pilot study. J. Dairy Sci..

[53] Iuliano S., Hare D.L., Vogrin S., Poon S., Robbins J., French C. (2024). Consumption of dairy foods to achieve recommended levels for older adults has no deleterious effects on serum lipids. Nutr. Metab. Cardiovasc. Dis..

[54] Appel L.J., Moore T.J., Obarzanek E., Vollmer W.M., Svetkey L.P., Sacks F.M. (1997). A clinical trial of the effects of dietary patterns on blood pressure. N. Engl. J. Med..

[55] Ndanuko R.N., Tapsell L.C., Charlton K.E., Neale E.P., Batterham M.J. (2016). Dietary patterns and blood pressure in adults: a systematic review and meta-analysis of randomized controlled trials. Adv. Nutr..

[56] Chiavaroli L., Viguiliouk E., Nishi S.K., Blanco Mejia S., Rahelić D., Kahleová H. (2019). DASH dietary pattern and cardiometabolic outcomes: an umbrella review of systematic reviews and meta-analyses. Nutrients.

[57] Sacks F.M., Svetkey L.P., Vollmer W.M., Appel L.J., Bray G.A., Harsha D. (2001). Effects on blood pressure of reduced dietary sodium and the Dietary Approaches to Stop Hypertension (DASH) diet. DASH-Sodium Collaborative Research Group. N. Engl. J. Med..

[58] Maki K.C., Rains T.M., Schild A.L., Dicklin M.R., Park K.M., Lawless A.L. (2013). Effects of low-fat dairy intake on blood pressure, endothelial function, and lipoprotein lipids in subjects with prehypertension or stage 1 hypertension, Vasc. Health Risk Manag..

[59] Sandby K., Magkos F., Chabanova E., Petersen E.T., Krarup T., Bertram H.C. (2024). The effect of dairy products on liver fat and metabolic risk markers in males with abdominal obesity - a four-arm randomized controlled trial. Clin. Nutr..

[60] Bellikci-Koyu E., Sarer-Yurekli B.P., Karagozlu C., Aydin-Kose F., Ozgen A.G., Buyuktuncer Z. (2022). Probiotic kefir consumption improves serum apolipoprotein A1 levels in metabolic syndrome patients: a randomized controlled clinical trial. Nutr. Res..

[61] Labonté M., Cyr A., Abdullah M.M., Lépine M.C., Vohl M.C., Jones P. (2014). Dairy product consumption has no impact on biomarkers of inflammation among men and women with low-grade systemic inflammation. J. Nutr..

[62] Dugan C.E., Aguilar D., Park Y.K., Lee J.Y., Fernandez M.L. (2016). Dairy consumption lowers systemic inflammation and liver enzymes in typically low-dairy consumers with clinical characteristics of metabolic syndrome. J. Am. Coll. Nutr..

[63] Rancourt-Bouchard M., Gigleux I., Guay V., Charest A., Saint-Gelais D., Vuillemard J.C. (2020). Effects of regular-fat and low-fat dairy consumption on daytime ambulatory blood pressure and other cardiometabolic risk factors: a randomized controlled feeding trial. Am. J. Clin. Nutr..

[64] Forouhi N.G., Koulman A., Sharp S.J., Imamura F., Kröger J., Schulze M.B. (2014). Differences in the prospective association between individual plasma phospholipid saturated fatty acids and incident type 2 diabetes: the EPIC-InterAct case-cohort study. Lancet Diabetes Endocrinol.

[65] Xu Y., Dugan M.E.R., Mapiye C., Vahmani P. (2023). Health effects of ruminant trans fatty acids with emphasis on type 2 diabetes. Front. Anim. Sci..

[66] Mozaffarian D., de Oliveira Otto M.C., Lemaitre R.N., Fretts A.M., Hotamisligil G., Tsai M.Y. (2013). trans-Palmitoleic acid, other dairy fat biomarkers, and incident diabetes: the Multi-Ethnic Study of Atherosclerosis (MESA). Am. J. Clin. Nutr..

[67] Tardiolo G., Di Salvo E., Tringali S., Bartolomeo G., Genovese C., Furfaro M.E. (2025). Ripening-associated changes in fatty acid composition and nutritional indices in Caciocavallo Silano PDO cheese. Foods.

[68] Bethancourt H.J., Schmidt K.A., Cromer G., Burhans M.S., Kuzma J.N., Hagman D.K. (2022). Assessing the validity of plasma phospholipid fatty acids as biomarkers of dairy fat intake using data from a randomized controlled intervention trial. Am. J. Clin. Nutr..

