# Supplementary material for: A Randomized Controlled Trial to Evaluate the Effects of Full-Fat and Low-Fat Fermented Dairy Foods on Markers of Cardiometabolic Health in Adults with Overweight or Obesity and Elevated Fasting Triglycerides
Source: Curr Dev Nutr. 2026 Aug 14;10(9):109516. doi: 10.1016/j.cdnut.2026.109516 (PMC13571445; doi:10.1016/j.cdnut.2026.109516)
Supplement: Supplementary file 1 [file mmc1.docx]

**Manuscript Title:** A randomized controlled trial to evaluate the effects of full-fat and low-fat fermented dairy foods on markers of cardiometabolic health in adults with overweight or obesity and elevated fasting triglycerides

**First Author:** Kevin C. Maki, PhD

**Supplementary Table 1.** List of principal investigators and sites

Rupal Trivedi, MD

Great Lakes Clinical Trials

(Andersonville) Chicago, IL & Gurnee, IL

Indika Edirisinghe, PhD

Illinois Institute of Technology—Center for Nutrition Research

Chicago, IL

Miguel Trujillo, DO

Excellence Medical & Research

Miami, Gardens, FL

Nana Gletsu-Miller, PhD

Indiana University—Nutrition and Exercise Research Center

Bloomington, IN &

Indiana Clinical and Translational Sciences Institute

Indianapolis, IN

**Supplementary Table 2.** Demographic and baseline characteristics of the per-protocol population (N = 98)^1^

|  | **Full-Fat** | **Low-Fat** | **Control** |
| --- | --- | --- | --- |
|  | *n = 33* | *n = 33* | *n = 32* |
| Age, years^2^ | 41.3 (13.6) | 49.0 (11.2) | 46.3 (14.3) |
| Gender |  |  |  |
| *Male, n (%)* | 21 (63.6%) | 17 (51.5%) | 16 (50.0%) |
| *Female, n (%)* | 12 (36.4%) | 16 (48.5%) | 16 (50.0%) |
| Race |  |  |  |
| *Asian, n (%)* | 5 (15.2%) | 1 (3.0%) | 2 (6.2%) |
| *Black/African American, n (%)* | 3 (9.1%) | 3 (9.1%) | -- |
| *Other, n (%)* | -- | -- | 1 (3.1%) |
| *White, n (%)* | 25 (75.8%) | 29 (87.9%) | 29 (90.6%) |
| Ethnicity |  |  |  |
| *Hispanic/Latino, n (%)* | 18 (54.5%) | 18 (54.5%) | 19 (59.4%) |
| *Not Hispanic/Latino, n (%)* | 15 (45.5%) | 15 (45.5%) | 13 (40.6%) |
| **Mean (SD)** |  |  |  |
| Height, cm | 168 (11.4) | 165 (10.4) | 166 (9.96) |
| Weight, kg | 87.9 (18.9) | 88.4 (16.3) | 85.6 (13.0) |
| Body Mass Index, kg/m^2^ | 30.9 (4.49) | 32.5 (3.92) | 31.1 (4.58) |
| Waist Circumference, cm | 101 (15.1) | 103 (15.3) | 104 (10.8) |
| Systolic Blood Pressure, mm Hg | 119 (7.60) | 121 (7.99) | 123 (11.3) |
| Diastolic Blood Pressure, mm Hg | 76.1 (6.19) | 77.9 (7.01) | 77.6 (7.41) |
| Heart Rate, bpm | 74.0 (7.04) | 71.8 (7.77) | 72.1 (7.07) |

^1^Age, weight, waist circumference, body mass index, blood pressures and heart rate were assessed at baseline. All other data were assessed at the screening visit.

^2^Years of age is provided as mean (standard deviation)

Abbreviations: bpm, beats per minute; SD, standard deviation

**Supplemental Table 3.** Frequency of treatment-emergent adverse events and number of participants with at least one treatment-emergent adverse event (safety, N = 110)

|  | ***n*** | **Number of TEAEs**^1^ | **Relationship of TEAE to Product**^2^ | **Number of Participants with at least 1 TEAE** | **P-Value^3^** |
| --- | --- | --- | --- | --- | --- |
| Control | 36 | 7 | Probably = 2; Unlikely = 3; Not Related = 2 | 4 (11.1%) | 0.711 |
| Low-Fat | 37 | 4 | Probably = 0; Unlikely = 1; Not Related = 3 | 3 (8.11%) |  |
| Full-Fat | 37 | 0 | Probably = 0; Unlikely = 0; Not Related = 0 | 0 (0.00%) |  |

^1^TEAE is defined as an adverse event that started during or after the first consumption of study product. Any adverse events that occurred after informed consent and prior to first consumption of study product is considered medical history.

^2^In the control group, two events were judged to be “probably” related to the non-dairy control foods. In one event, the participant temporarily experienced moderate heartburn but recovered fully, and in the other event, the participant had mild bloating and recovered fully.
^3^P-value tested the null hypothesis that the distribution of participants with at least one TEAE is the same for each product group, using a chi-square test.

Abbreviations: TEAE, treatment-emergent adverse event

**Supplemental Table 4.** Correlation of waist circumference with insulin sensitivity and select markers of cardiometabolic health in the intent-to-treat population (N = 110)^1^

|  | **Waist Circumference Baseline** | | **Waist Circumference Ratio Change** | |
| --- | --- | --- | --- | --- |
|  | *Pearson’s r-value^2^* | *P-value^3^* | *Pearson’s r-value^2^* | *P-value^3^* |
| **MISI** |  |  |  |  |
| Overall | -0.272 | **0.004** | 0.110 | 0.271 |
| Control | -0.132 | 0.443 | -0.102 | 0.566 |
| Low-Fat | -0.341 | **0.039** | 0.160 | 0.367 |
| Full-Fat | -0.373 | **0.025** | 0.125 | 0.481 |
| **HOMA-IR** | | | | |
| Overall | 0.232 | **0.015** | -0.034 | 0.726 |
| Control | 0.202 | 0.238 | 0.191 | 0.272 |
| Low-Fat | 0.230 | 0.170 | -0.088 | 0.614 |
| Full-Fat | 0.285 | 0.088 | -0.026 | 0.880 |
| **LDL Cholesterol** | | | | |
| Overall | -0.118 | 0.218 | -0.044 | 0.652 |
| Control | -0.152 | 0.377 | -0.126 | 0.470 |
| Low-Fat | -0.134 | 0.430 | 0.147 | 0.399 |
| Full-Fat | -0.043 | 0.799 | -0.070 | 0.686 |
| **HDL Cholesterol** | | | | |
| Overall | -0.284 | **0.003** | 0.020 | 0.835 |
| Control | -0.249 | 0.144 | -0.009 | 0.960 |
| Low-Fat | -0.348 | **0.035** | 0.064 | 0.716 |
| Full-Fat | -0.291 | 0.081 | 0.028 | 0.869 |
| **Non-HDL Cholesterol** | | | | |
| Overall | -0.066 | 0.494 | -0.011 | 0.908 |
| Control | -0.071 | 0.682 | -0.167 | 0.339 |
| Low-Fat | -0.107 | 0.529 | 0.153 | 0.381 |
| Full-Fat | -0.013 | 0.940 | -0.001 | 0.995 |
| **Triglycerides** | | | | |
| Overall | 0.098 | 0.309 | 0.086 | 0.381 |
| Control | 0.119 | 0.489 | -0.052 | 0.767 |
| Low-Fat | 0.023 | 0.891 | 0.122 | 0.484 |
| Full-Fat | 0.133 | 0.433 | 0.128 | 0.457 |
| **Apolipoprotein B** | | | | |
| Overall | 0.015 | 0.879 | -0.002 | 0.986 |
| Control | -0.016 | 0.929 | -0.019 | 0.918 |
| Low-Fat | 0.032 | 0.858 | 0.233 | 0.207 |
| Full-Fat | 0.055 | 0.752 | -0.104 | 0.557 |
| **Small Dense LDL Cholesterol** | | | | |
| Overall | 0.104 | 0.282 | -0.345 | **<0.001** |
| Control | -0.150 | 0.389 | -0.417 | **0.014** |
| Low-Fat | 0.290 | 0.087 | -0.119 | 0.502 |
| Full-Fat | 0.265 | 0.113 | -0.492 | **0.002** |

^1^N = 110 for overall group; n = 36 for non-dairy control; n = 37 for low-fat fermented dairy; and n = 37 for full-fat fermented dairy.

^2^Pearson correlations coefficients were examined separately for natural log transformed baseline values and for the natural log transformed ratio change from baseline to week 12. Ratio change was computed at the participant level as the week 12 value divided by the baseline value. Correlations were generated using observed values only.

^3^P-value tested the null hypothesis that there was no linear relationship between waist circumference and the cardiometabolic variable between each product type.

Abbreviations: HOMA-IR, homeostatic model assessment for insulin resistance, MISI, Matsuda insulin sensitivity index.
