# Supplementary material for: A Randomized Controlled Trial to Evaluate the Effects of Full-Fat and Low-Fat Fermented Dairy Foods on Markers of Cardiometabolic Health in Adults with Overweight or Obesity and Elevated Fasting Triglycerides
Source: Curr Dev Nutr. 2026 Aug 14;10(9):109516. doi: 10.1016/j.cdnut.2026.109516 (PMC13571445; doi:10.1016/j.cdnut.2026.109516)
Supplement: Supplementary file 2 [file mmc2.docx]

**Manuscript Title:** A randomized controlled trial to evaluate the effects of full-fat and low-fat fermented dairy foods on markers of cardiometabolic health in adults with overweight or obesity and elevated fasting triglycerides

**First Author:** Kevin C. Maki, PhD

**Supplemental Material: Calculations for Derivation of Study Outcome Measures**

*Matsuda index of insulin sensitivity (MISI):*

MISI is calculated from the liquid meal tolerance test (LMTT) and is validated index of whole-body insulin sensitivity (1-3). MISI was computed using units of µIU/mL for insulin and mg/dL for glucose and was computed as follows (4):

$$MISI= \frac{10,000}{(fasting insulin*fasting glucose*mean insulin*mean glucose)^0.5}$$

The mean insulin and mean glucose were computed as the total area under the curve from 0-120 min (total _AUC0-120 min_) divided by 120 min, using only the t = 0, 60, 120 min values. Total AUC_0-120 min_ was computed as described by Brouns et al. (2005) (5).

*Insulin secretion index (ISI):*

ISI was computed using units of µIU/mL for insulin and mg/dL for glucose. ISI was calculated as follows, where total AUC_0-120 min_ was computed as described by Brouns et al. (2005) (5).

$$ISI= \frac{{insulin total AUC}_{0-120 min}}{{glucose total AUC}_{0-120 min}}$$

*Disposition index (DI):*

DI was computed from the LMTT using units of µU/mL for insulin and mg/dL for glucose and was considered a key carbohydrate metabolism marker. DI was calculated as:

$$DI=ISI*MISI$$

*Homeostatic model assessment for beta-cell function (HOMA-B) & homeostatic model assessment for insulin resistance (HOMA-IR):*

HOMA-IR and HOMA-B are considered supportive markers of carbohydrate metabolism. Both were computed using units of µIU/mL for insulin and mg/dL for glucose and calculated as follows:

$$HOMA-B= \frac{20*fasting insulin}{fasting glucose-63}$$

$$HOMA-IR=\frac{fasting insulin*fasting glucose}{405}$$

*Cohen’s d:*

Cohen's d is a standardized effect size that measures the difference between two group means in terms of standard deviations, rather than raw units. Cohen’s d was computed as:

$$d=\frac{{LSM}_{condition1}-{LSM}_{condition2}}{{SD}_{pooled}}$$

where LSM is the least squares mean difference and ${SD}_{pooled}$ is the pooled standard error computed from the standard errors of the LSMs.

**Supplemental Material References**

1.	Muniyappa R, Madan R, Varghese RT. Assessing Insulin Sensitivity and Resistance in Humans. South Dartmouth, MA, 2024.

2.	Moriyama K. Mini-review on insulin resistance assessment: Advances in surrogate indices and clinical applications. World J Clin Cases. 2025;13(29):108380. doi: 10.12998/wjcc.v13.i29.108380.

3.	Maki KC, Kelley KM, Lawless AL, Hubacher RL, Schild AL, Dicklin MR, Rains TM. Validation of insulin sensitivity and secretion indices derived from the liquid meal tolerance test. Diabetes Technol Ther. 2011;13(6):661-6. doi: 10.1089/dia.2010.0240.

4.	Matsuda M, DeFronzo RA. Insulin sensitivity indices obtained from oral glucose tolerance testing: comparison with the euglycemic insulin clamp. Diabetes Care. 1999;22(9):1462-70. doi: 10.2337/diacare.22.9.1462.

5.	Brouns F, Bjorck I, Frayn KN, Gibbs AL, Lang V, Slama G, Wolever TMS. Glycaemic index methodology. Nutrition Research Reviews. 2005;18(1):145-71. doi: 10.1079/NRR2005100.
